# Synthesis of 1,2-divinylcyclopropanes by metal-catalyzed cyclopropanation of 1,3-dienes with cyclopropenes as vinyl carbene precursors

**DOI:** 10.3762/bjoc.15.25

**Published:** 2019-01-30

**Authors:** Jesús González, Alba de la Fuente, María J González, Laura Díez de Tejada, Luis A López, Rubén Vicente

**Affiliations:** 1 Departmento de Química Orgánica e Inorgánica e Instituto Universitario de Química Organometálica "Enrique Moles", Universidad de Oviedo, Julián Clavería 8, 33006-Oviedo, Spain

**Keywords:** cyclopropanation, cyclopropenes, dienes, divinylcyclopropanes, transition metal catalysis

## Abstract

The synthesis of 1,2-divinylcyclopropanes by the reaction of cyclopropenes with 1,3-dienes is reported. The process relies on the ability of ZnCl_2_ or [Rh_2_(OAc)_4_] to generate metal–vinyl carbene intermediates from cyclopropenes, which effect cyclopropanation of 1,3-dienes. Most of the reactions proceeded in reasonable yields while the diastereoselectivity strongly depends on the structure of the diene. An example of an intramolecular process as well as the use of furan and 1,4-cyclohexadiene as dienes are also reported.

## Introduction

**Scheme 1:**
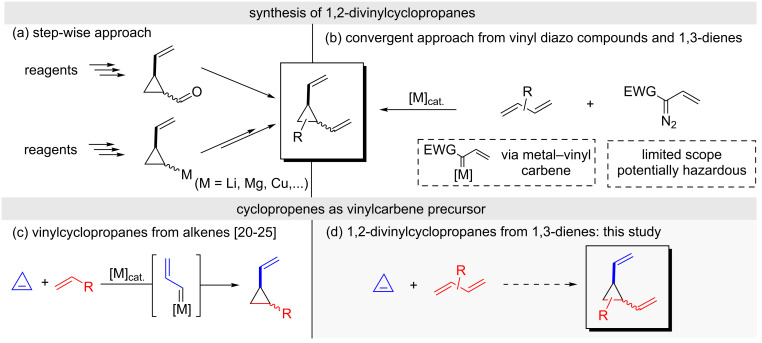
Typical syntheses of 1,2-divinylcyclopropanes and rationale hypothesis for their syntheses from cyclopropenes and 1,3-dienes.

Far from being considered exotic molecules, cyclopropane derivatives constitute an interesting class of compounds. Indeed, far over 4000 natural products bearing a cyclopropane ring have been discovered [1–3], and cyclopropane-containing molecules are recurrent in medicinal chemistry [4–6]. Likewise, due to its unique structure and bond properties, cyclopropanes have exclusive yet useful synthetic utilities [7], which are closely connected with the substitution pattern. For instance, the presence of vinyl groups directly attached to a cyclopropane ring allows sigmatropic rearrangements leading to odd-numbered carbocyclic derivatives [8]. In this sense, seven-membered carbocycles, namely 1,4-cycloheptadienes, can be forthrightly prepared from *cis*- or *trans*-1,2-divinylcyclopropanes through a Cope rearrangement [8–9]. The potential of this type of cyclopropanes contrasts with the existence of few straightforward routes for their syntheses. Typical methods rely on the use of reagents containing the required cyclopropane ring, which involve multistep sequences for the installation of adequate functionalization. Thus, Wittig-type olefination with cyclopropanecarboxaldehydes [10] or reactions of metallated vinylcyclopropanes with suitable electrophiles are commonly employed (Scheme 1a) [11–13]. In a more convergent approach where the cyclopropane ring is created at the last stage, divinylcyclopropanes can be prepared by cyclopropanation of 1,3-dienes with metal–vinyl carbenes generated from vinyldiazoacetates (Scheme 1b) [14–16]. This reaction has been fruitfully exploited, although it is inherently limited by the restricted availability of potentially explosive diazo compounds. Consequently, the use of alternative vinyl carbene precursors is highly desirable to expand the accessibility to 1,2-divinylcyclopropanes [17–19]. In this regard, cyclopropenes have demonstrated to be suitable precursors of metal–vinyl carbenes [20–21], which can be easily trapped with alkenes [22–25]. Our recent studies showed that simple ZnCl_2_ could be used to generate the corresponding zinc–vinyl carbene to efficiently prepare vinylcyclopropane derivatives with a remarkable broad scope (Scheme 1c) [26]. In view of these precedents, we decided to study the feasibility of this reaction in the synthesis of 1,2-divinylcyclopropanes by using 1,3-dienes as trapping reagents (Scheme 1d). Herein, we present the results of this study.

## Results and Discussion

**Scheme 2:**
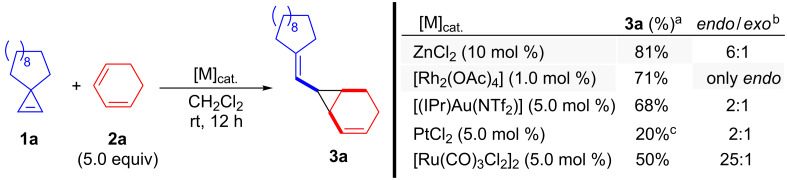
Synthesis of 1,2-divinylcyclopropane **3a**: Optimization studies. ^a^Isolated yield. ^b^Determined by ^1^H NMR on the reaction crude mixture (unaltered after purification). ^c^Estimated by ^1^H NMR. (IPr = 1,3-bis(2,6-diisopropylphenylimidazol-2-ylidene).

At the outset, the reaction of cyclopropene **1a** and freshly distilled 1,3-cyclohexadiene (**2a**, 5.0 equiv) in the presence of ZnCl_2_ as catalyst was performed under the reaction conditions previously employed for simple alkenes, namely ZnCl_2_ 10 mol %, CH_2_Cl_2_, at ambient temperature (Scheme 2) [26]. Pleasantly, 1,2-divinylcyclopropane **3a** was obtained in good yield (81%) with moderate *endo* (*syn*) selectivity (*endo*/*exo* = 6:1). It should be indicated that the preference for the *endo* isomer has already been observed in related reactions [17]. Other metals capable of promoting both carbene generation from cyclopropenes and cyclopropanation reactions were also evaluated. Interestingly, when [Rh_2_(OAc)_4_] (1.0 mol %) was employed, compound **3a** was isolated in 71% yield, a slightly lower value when compared to ZnCl_2_, but more importantly, with complete *endo* (*syn*) selectivity. The use of gold, platinum or ruthenium catalysts showed poorer results with respect to reaction yield and *endo*/*exo* (*syn*/*anti*) selectivity, as indicated in Scheme 2.

**Scheme 3:**
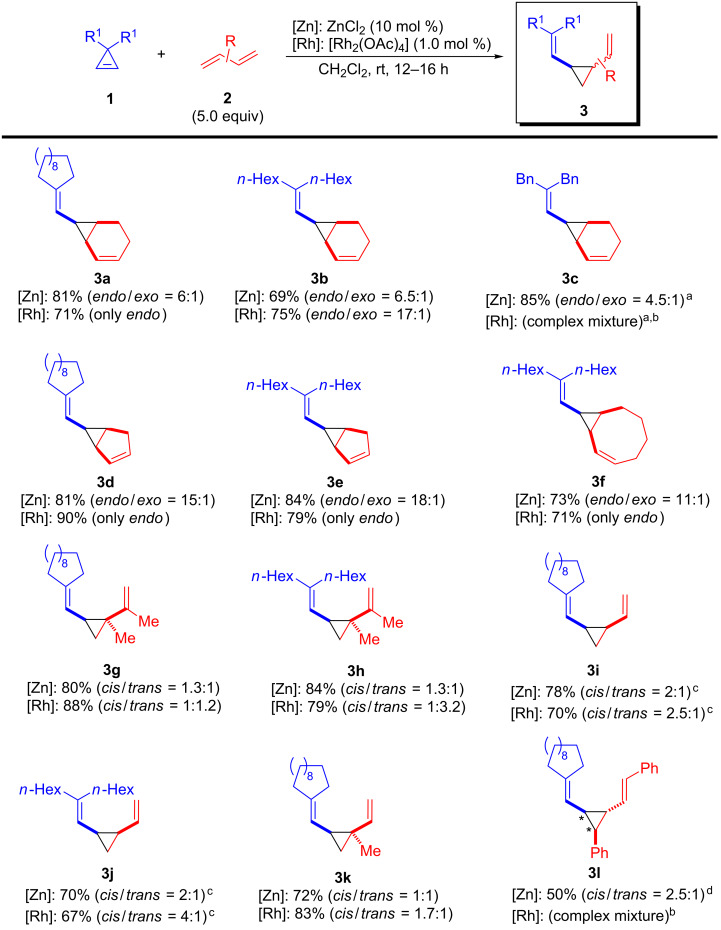
Synthesis of 1,2-divinylcyclopropanes **3** from cyclopropenes **1** and unbiased 1,3-dienes **2**: Scope. (Yields of isolated products, diastereoisomeric ratios were determined by ^1^H NMR). ^a^At 50 °C. ^b^**1** was completely consumed. ^c^1,3-Butadiene was used in large excess (ca. 0.5 mL condensed prior the reaction). ^d^*cis* relationship is referred to substituents labelled with an asterisk.

Considering these results, the scope of the reaction with unbiased 1,3-dienes was next investigated using both ZnCl_2_, as it provided the best yield being the most inexpensive catalyst, and [Rh_2_(OAc)_4_], since it delivered the best result in terms of selectivity. The results are summarized in Scheme 3.

Using 1,3-cyclohexadiene (**2a**), the analogous reaction was accomplished with 3,3-dihexyl- (**1b**) and 3,3-dibenzylcyclopropene (**1c**). Thus, divinylcyclopropane **3b** was obtained in reasonable yields with both catalysts (69% Zn and 75% Rh), albeit better selectivity was obtained again with rhodium(II) catalyst (*endo*/*exo* = 17:1). In contrast, the reaction with **1c** led to the corresponding cyclopropane **3c** only when ZnCl_2_ was used as catalyst (85%, *endo*/*exo* = 4.5:1) at slightly higher temperature (50 °C). In this case, [Rh_2_(OAc)_4_] completely failed under different reaction conditions leading inevitably to degradation of starting cyclopropene. Other cyclic 1,3-dienes were then evaluated. For instance, the use of cyclopentadiene in the reaction with **1a** and **1b** enabled the preparation of divinylcyclopropanes **3d**,**e** in good yields. Both catalysts provided similar yields, while complete *endo* selectivity was only reached with [Rh_2_(OAc)_4_]. Interestingly, the use of 1,3-cyclooctadiene led to the formation of compound **3f** (Zn: 73%, *endo*/*exo* = 11:1; Rh: 71%, only *endo*), in which the 1,2-divinylcyclopropane moiety is embedded within a bicyclo[6.1.0]nonane core. Then, we studied the reaction with various representative unbiased acyclic 1,3-dienes. Unsurprisingly, these substrates could be easily converted into the corresponding 1,2-divinylcyclopropanes but with very low selectivities. For instance, when 2,3-dimethyl-1,3-butadiene was employed with cyclopropenes **1a**,**b**, the corresponding 1,2-divinylcyclopropanes **3g**,**h** were prepared in good yields regardless of the catalyst employed. However, **3g**,**h** were obtained as an almost equimolar mixture of *cis/trans* diastereoisomers (*cis* refers to both vinyl substituents). Even though the lack of selectivity was already noticed by Uemura and co-workers in related reactions [17], we attempted the reaction with other zinc salts or rhodium(II) carboxylates as catalysts. Unfortunately, these experiments were futile and led to similar low selectivities. Besides, gaseous 1,3-butadiene could be also employed, as demonstrated by the preparation of compounds **3i**,**j**, which were obtained in moderate yields and low *cis* selectivities. The reaction with isoprene showed a remarkable selectivity for the most substituted alkene, allowing the synthesis of 1,2-divinylcyclopropane **3k** within the typical range of yields and *cis/trans* selectivities. Finally, 1,2,3-trisubstituted divinylcyclopropane **3l** was prepared in 50% (*cis*/*trans* = 2.5:1) from cyclopropene **1a** and (1*E*,3*E*)-1,4-diphenylbuta-1,3-diene through a stereoselective reaction. Once more, simple ZnCl_2_ was particularly effective for this reaction in sharp contrast to the incompetence of [Rh_2_(OAc)_4_].

It should be noticed that 1,2-divinylcyclopropanes **3a**–**l** did not undergo [3,3]-Cope rearrangements under the reaction conditions. Moreover, when pure *endo*-**3e** was refluxed in toluene (24 h) partial isomerization was observed (*endo*/*exo* = 1:1.6). This behavior can be attributed to the high substitution at one of the alkenes, which might lead to sterically overcrowded transition states required for the rearrangement [27]. Besides, the diastereoisomeric mixture of **3j** suffered complete degradation under the same reaction conditions.

**Scheme 4:**
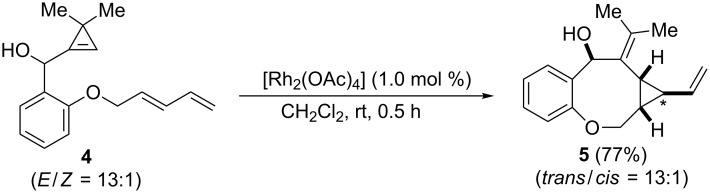
Rh-catalyzed intramolecular cyclopropanation with dienylcyclopropene **4** (the *trans*/*cis* ratio is related to the C-atom labelled with an asterisk).

In recent years, Cossy and co-workers elegantly prepared various bicyclo[*n*.1.0] derivatives through intramolecular cyclopropanation reactions using adequately decorated 1,*n*-cyclopropenenes [24]. Hence, we preliminarily explored the feasibility of an analogous reaction with 1,3-dienes preparing dienylcyclopropene **4**. While treatment of **4** with ZnCl_2_ led to complex mixtures, likely due to the Lewis acid sensitivity of the benzylic cyclopropenylcarbinol moiety, the use of [Rh_2_(OAc)_4_] (1.0 mol %, CH_2_Cl_2_, rt) led to the tricyclic compound **5** in good yield (77%) in a stereoctive manner (Scheme 4).

**Scheme 5:**
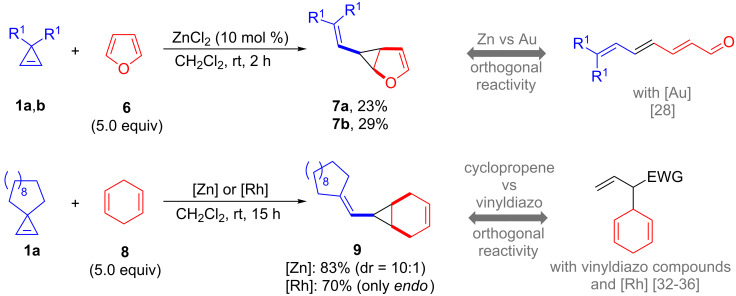
Zn- or Rh-catalyzed reactions of cyclopropenes **1** with furan (**6**) and 1,4-cyclohexadiene (**8**) and comparison with related processes (**1b**, R^1^ = *n*-Hex).

Finally, we were curious to study the reactivity of metal–vinyl carbenes generated from 3,3-disubstituted cyclopropenes with some particular dienes (Scheme 5). Interestingly, we found that the reaction of cyclopropenes **1a**,**b** with furan (**6**) using ZnCl_2_ as catalyst allowed the isolation of diastereomerically pure *endo*-**7a**,**b** oxabicycles showing the 1,2-divinylcyclopropane moiety. In contrast, [Rh_2_(OAc)_4_] afforded complex reaction mixtures. In spite of the modest yields, this zinc-catalyzed reaction deserves some comments as it constitutes a rare example of isolation of these structures. Indeed, Lee and co-worker found that the corresponding gold-catalyzed reaction leads to ring-opened products through a facile oxy-Cope-rearrangement [28]. Moreover, these structures are also not accessible with metal–vinyl carbenes generated from vinyldiazo compounds, which led again to oxy-Cope rearranged or [4 + 3]-cycloaddition products using rhodium catalysts [14,28–30], or to a C2-allylation of furan with gold catalysts [31]. Finally, to compare the reactivity of cyclopropenes and vinyldiazo compounds, we probed the reaction of **1a** with 1,4-cyclohexadiene (**8**). Under otherwise identical reaction conditions, both ZnCl_2_ and [Rh_2_(OAc)_4_] yielded exclusively cyclopropane **9** in practical yields and *endo* selectivity. This reaction outcome differs from the comparable reactions with vinyldiazo compounds and rhodium(II) catalysts, which preferentially undergo C–H allylic insertions [31–35].

## Conclusion

In summary, we have described a straightforward method for the synthesis of 1,2-divinylcyclopropane derivatives by cyclopropanation reactions using unbiased 1,3-dienes and cyclopropenes, which served as metal–vinyl carbene precursors. The use of cyclic dienes allowed the synthesis of the corresponding 1,2-divinylcyclopropanes with good-to-complete *endo* selectivity. In contrast, acyclic dienes were also efficiently converted into the expected cyclopropanes but with low *cis*/*trans* selectivities. In general, simple ZnCl_2_ and [Rh_2_(OAc)_4_] proved to be adequate catalysts. An intramolecular version of this reaction is also reported. Finally, the use of other dienes, such as furan and 1,4-cyclohexadiene was explored showing interesting reaction outcomes that are complementary to previous related reports. Subsequent studies to expand the scope of this cyclopropanation reaction to biased 1,3-dienes as well as to increase selectivities are currently ongoing.

## Supporting Information

File 1 Experimental details as well as ^1^H and ^13^C NMR spectra of new compounds.
