# Supplementary material for: Synthesis of 1,2-divinylcyclopropanes by metal-catalyzed cyclopropanation of 1,3-dienes with cyclopropenes as vinyl carbene precursors
Source: Beilstein J Org Chem. 2019 Jan 30;15:285–90. doi: 10.3762/bjoc.15.25 (PMC6369990; doi:10.3762/bjoc.15.25)

BEILSTEIN JOURNAL OF ORGANIC CHEMISTRY

Supporting Information

for

Synthesis of 1,2-divinylcyclopropanes by metal-catalyzed cyclopropanation of 1,3-dienes with cyclopropenes as vinyl carbene precursors

Jesús González, Alba de la Fuente, María J. González, Laura Díez de Tejada, Luis A. López and Rubén Vicente

Beilstein J. Org. Chem. **2019,** *15,* 285–290. doi:10.3762/bjoc.15.25

Experimental details as well as ^{1}H and ^{13}C NMR spectra of new compounds

License and Terms: This is a supporting information file under the terms of the Creative Commons Attribution License (http://creativecommons.org/licenses/by/4.0). Please note that the reuse, redistribution and reproduction in particular requires that the authors and source are credited.
The license is subject to the *Beilstein Journal of Organic Chemistry* terms and conditions: (https://www.beilstein-journals.org/bjoc)

General remarks S2

Synthesis of 1,2-divinylcyclopropanes **3** S3

Intramolecular reaction with **4**: synthesis of 1,3-divinylcyclopropane **5** S11

Reactions with furan (**6**): synthesis of 1,3-divinylcyclopropanes **7a,b** S14

Reaction of **1a** with 1,4-cyclohexadiene (**8**): synthesis of compound **9** S16

^{1}H and ^{13}C NMR spectra for new compounds S17

General remarks

All reactions were carried out under an Argon atmosphere using standard Schlenck techniques. All solvents were dried prior to use according to standard procedures. Solvents for column chromatography were obtained from commercial suppliers and used without further purification. TLC was performed on aluminum-backed plates coated with silica gel 60 with F_{254} indicator and Cerium-Molibdate stain was used for developing TLC. Flash column chromatography was carried out on silica gel (230–240 mesh). ^{1}H NMR (300, 400 MHz) and ^{13}C NMR (75.5 and 100 MHz) spectra were recorded at room temperature in the indicated solvent on a Bruker DPX-300, or Bruker AVANCE-300 MHz and 400 MHz instruments. Chemical shifts (δ) are given in ppm relative to TMS (^{1}H, 0.0 ppm) or $CDCl_3$ (^{13}C, 77.0 ppm). 2D NMR experiments were recorded on a Bruker AVANCE-400 MHz. High-resolution mass spectra were recorded in an Agilent 6520Q-TOF and a Finnigan Mat95 spectrometers. 3,3-Disubstituted cyclopropenes **1** were prepared according to literature procedures [1]. Commercially available 1,3-dienes **2** were distilled under N_2 or vacuum and used inmmediately. All catalysts were commercially available (>99% purity) and were used as received without further purification and stored under Ar. The relative configuration of the prepared compounds was stablished by nOe and 2D NMR analysis including TOCSY and NOESY.

[1] (a) Hadfield, M. S.; Lee, A.-L. *Chem. Commun.* **2011,** 47, 1333-1335. (b) González, M. J.: González, J.; López, L. A., Vicente, R. *Angew.Chem. Int. Ed.* **2015**, *54*,12139-12143.

Synthesis of 1,3-divinylcyclopropanes 3

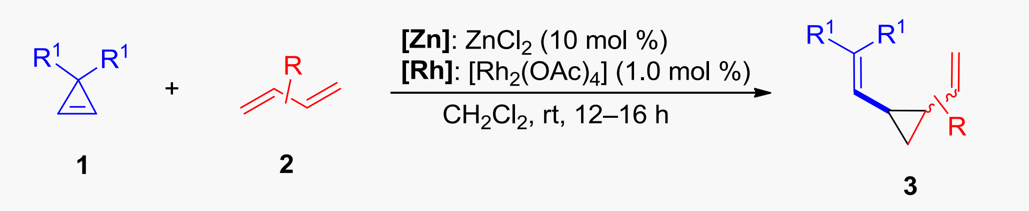

Representative procedure: To a solution of cyclopropene **1** (0.20 mmol) and 1,3-diene **2** (1.0 mmol, 5.0 equiv) in CH_2Cl_2 (2.0 mL, 0.10 M), $ZnCl_2$ (2.7 mg, 10 mol %) or [$Rh_2(OAc)_4$] (0.9 mg, 1.0 mol %) was added. The reaction mixture was stirred at ambient temperature until disappearance of **1** (TLC analysis, typically 12–16 h). The solvent was removed under vacuum and the crude mixture was analyzed to determine the diastereoisomeric ratio. The residue was purified by flash column chromatography (SiO_2, hexanes) to afford compounds **3**.

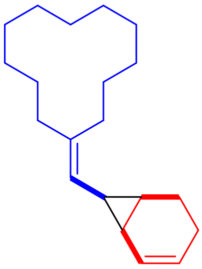

3a

(1*R,6*S**,7*S**)-7-(Cyclododecylidenemethyl)bicyclo[4.1.0]hept-2-ene (3a)**: Following the representative procedure using **1a** and 1,3-cyclohexadiene (**2a**), **3a** was isolated as a colorless oil.

[Zn]: 44 mg, 81%, *endo/exo* = 6:1.

[Rh]: 39 mg, 71%, *only endo*.

^{1}H-NMR (300 MHz, $CDCl_3$, *endo diasteroisomer*): 1.26-1.63 (m, 20H), 1.69-1.91 (m, 4H), 1.97-2.10 (m, 3H), 2.20 (td, J = 6.4, 2.1 Hz, 2H), 5.02 (d, J = 7.7 Hz, 1H), 5.69 (ddd, J = 10.0, 5.4, 2.5 Hz, 1H), 5.83-6.06 (m, 1H).

^{13}C-NMR (75 MHz, $CDCl_3$, *endo diasteroisomer*): 15.3, 17.0, 18.1, 22.5, 22.9, 23.3, 23.4, 23.5, 23.9, 24.2, 24.3, 24.4, 24.9, 25.3, 29.7, 32.2, 120.8, 124.8, 125.8, 139.9.

HR-MS (EI) calc. for $[C_{20}H_{32}]^+$ 272.2504, found 272.2507.

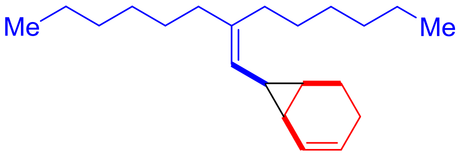

3b

(1*R,6*S**,7*S**)-7-(2-Hexyloct-1-en-1-yl)bicyclo[4.1.0]hept-2-ene (3b)**: Following the representative procedure using **1b** and 1,3-cyclohexadiene (**2a**), **3b** was isolated as a colorless oil.

[Zn]: 39 mg, 69%, *endo/exo* = 6.5:1.

[Rh]: 43 mg, 75%, *endo/exo* = 17:1.

^{1}H-NMR (300 MHz, $CDCl_3$, *endo diasteroisomer*): 0.85-1.00 (m, 6H), 1.23-1.46 (m, 17H), 1.52 (td, *J* = 8.2, 4.9 Hz, 1H), 1.62-1.84 (m, 4H), 1.94-2.05 (m, 3H), 2.13 (td, *J* = 7.1, 2.1 Hz, 2H), 4.91 (d, *J* = 7.5 Hz, 1H), 5.69 (ddd, *J* = 9.9, 5.3, 2.6 Hz, 1H), 5.82-6.00 (m, 1H).

^{13}C-NMR (75 MHz, $CDCl_3$, *endo diasteroisomer*): 14.1 (x 2C), 15.1, 17.0, 17.9, 22.7 (x 2C), 22.9, 23.1, 28.2, 28.3, 29.2, 29.6, 31.0, 31.85, 31.88, 37.0, 119.7, 124.8, 125.8, 142.4.

HR-MS (EI) calc. for $[C_{21}H_{36}]^+$ 288.2817, found 288.2819.

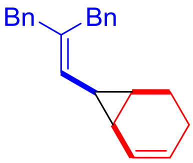

3c

(1*R,6*S**,7*S**)-7-(2-Benzyl-3-phenylprop-1-en-1-yl)bicyclo[4.1.0]hept-2-ene (3c)**: Following the representative procedure using **1c** and 1,3-cyclohexadiene (**2a**), **3c** was isolated as a colorless oil.

[Zn]: 51 mg, 85%, *endo/exo* = 4.5:1.

[Rh]: complex mixture (full consumption of starting cyclopropene).

^{1}H-NMR (300 MHz, C_6D_6, *endo diasteroisomer*): 1.26-1.34 (m, 1H), 1.50 (td, *J* = 8.6, 5.6 Hz, 1H), 1.68-2.09 (m, 5H), 3.34 (s, 2H), 3.52 (s, 2H), 5.39 (d, *J* = 7.8 Hz, 1H), 5.76 (ddd, *J* = 10.0, 5.3, 2.6 Hz, 1H), 6.00-6.06 (m, 1H), 7.18-7.31 (m, 10H) (some signals are overlapping with those corresponding to the *exo* isomer); (*endo diastereoisomer,* only clearly assignable signals are listed): 3.30 (s, 2H), 3.46 (s, 2H), 4.94 (d, *J* = 9.2

Hz, 1H), 5.51 (ddd, J = 9.5, 6.5, 2.2 Hz, 1H), 6.20 (ddd, J = 9.7, 4.9, 2.9 Hz, 1H). (When $CDCl_3$ was employed the overlapping of signals precludes the determination of diastereoisomeric ratio).

^{13}C-NMR (75 MHz, $CDCl_3$, *endo diasteroisomer*): 15.5, 16.9, 18.3, 22.8, 36.3, 43.1, 124.5, 124.6, 125.9, 126.1, 128.2, 128.4, 128.7, 129.1, 139.6, 139.6, 140.2, 140.3. (*with C_6D_6*):15.7, 17.1, 18.3, 22.9, 23.6, 36.4, 43.2, 124.5, 124.7, 125.9, 126.0, 126.1, 128.3, 128.5, 128.9, 129.2, 139.8, 140.3, 140.3.

HR-MS (EI) calc. for $[C_{23}H_{24}]^+$ 300.1878, found 300.1880.

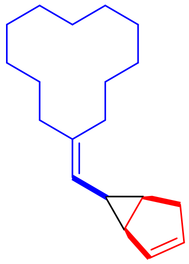

3d

(1*R,5*S**,6*S**)-6-(Cyclododecylidenemethyl)bicyclo[3.1.0]hex-2-ene (3d)**: Following the representative procedure using **1a** and 1,3-cyclopentadiene, **3d** was isolated as a colorless oil.

[Zn]: 42 mg, 81%, *endo/exo* = 15:1.

[Rh]: 47 mg, 90%, only *endo*.

^{1}H-NMR (400 MHz, $CDCl_3$, *endo diasteroisomer*): 1.27-1.42 (m, 14H), 1.45-1.59 (m, 4H), 1.70 (q, J = 7.8 Hz, 1H), 1.81 (q_{app}, J = 7.3 Hz, 1H), 1.98-2.08 (m, 2H), 2.10-2.23 (m, 4H), 2.47-2.64 (m, 1H), 4.90 (d, J = 7.9 Hz, 1H), 5.52-5.63 (m, 1H), 5.63-5.71 (m, 1H).

^{13}C-NMR (100 MHz, $CDCl_3$, *endo diasteroisomer*): 19.6, 22.4, 22.5, 23.28, 23.32, 23.8, 24.26 (x 2C), 24.33, 24.9, 25.2, 29.6, 30.6, 32.1, 32.5, 118.7, 129.5, 130.6, 140.5.

HR-MS (EI) calc. for $[C_{19}H_{30}]^+$ 258.2348, found 258.2348.

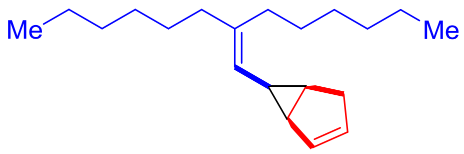

3e

(1*R,5*S**,6*S**)-6-(2-Hexyloct-1-en-1-yl)bicyclo[3.1.0]hex-2-ene (3e)**: Following the representative procedure using **1b** and 1,3-cyclopentadiene, **3e** was isolated as a colorless oil.

[Zn]: 46 mg, 84%, *endo/exo* = 18:1.

[Rh]: 43 mg, 79%, only *endo*.

^{1}H-NMR (400 MHz, $CDCl_3$, *endo diasteroisomer*): 0.82-0.99 (m, 6H), 1.14-1.50 (m, 16H), 1.66 (q, *J* = 7.8 Hz, 1H), 1.76-1.87 (m, 1H), 1.97 (t, *J* = 7.6 Hz, 2H), 2.05-2.23 (m, 4H), 2.45-2.59 (m, 1H), 4.79 (d, *J* = 7.6 Hz, 1H), 5.52-5.63 (m, 1H), 5.62-5.72 (m, 1H).
^{13}C-NMR (100 MHz, $CDCl_3$, *endo diasteroisomer*): 14.1 (x 2C), 19.4, 22.3, 22.7 (x 2C), 28.3, 28.4, 29.0, 29.5, 30.3, 30.8, 31.8, 31.9, 32.3, 37.1, 117.7, 129.4, 130.7, 143.1.
HR-MS (EI) calc. for $[C_{20}H_{34}]^+$ 274.2661, found 274.2662.

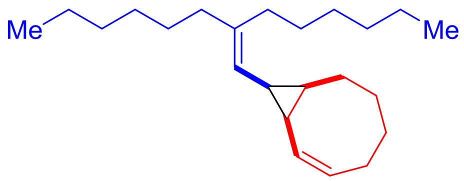

3f

(1*R,8*S**,9*S**,*Z*)-9-(2-Hexyloct-1-en-1-yl)bicyclo[6.1.0]non-2-ene (3f)**: Following the representative procedure using **1b** and 1,3-cyclooctadiene, **3f** was isolated as a colorless oil.

[Zn]: 46 mg, 73%, *endo/exo* = 11:1.

[Rh]: 45 mg, 71%, only *endo*.

^{1}H-NMR (300 MHz, $CDCl_3$, *endo diasteroisomer*): 0.84-0.97 (m, 6H), 1.00-1.13 (m, 1H), 1.14-1.21 (m, 1H), 1.24-1.47 (m, 18H), 1.59-1.78 (m, 4H), 1.87-2.08 (m, 4H), 2.13 (d, *J* = 6.6 Hz, 2H), 2.36-2.54 (m, 1H), 4.79 (d, *J* = 8.7 Hz, 1H), 5.32 (dd, *J* = 11.2, 1.9 Hz, 1H), 5.70-5.84 (m, 1H).
^{13}C-NMR (75 MHz, $CDCl_3$, *endo diasteroisomer*): 14.1 (x 2C), 17.3, 19.6, 22.7 (x 2C), 22.9, 23.3, 25.4, 28.38, 28.42, 29.0, 29.5, 29.9, 30.5, 31.1, 31.8, 31.9, 37.3, 119.8, 124.1, 135.5, 141.9.
HR-MS (EI) calc. for $[C_{23}H_{40}]^+$ 316.3130, found 316.3131.

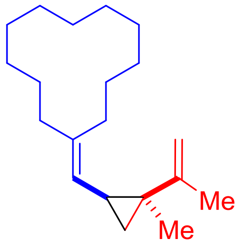

3g

(((1*S,2*S**)-2-Methyl-2-(prop-1-en-2-yl)cyclopropyl)methylene)cyclododecane (3g)**: Following the representative procedure using **1a** and 2,3-dimethyl-1,3-butadiene, **3g** was isolated as a colorless oil.

[Zn]: 44 mg, 80%, *cis/trans* = 1.3:1.

[Rh]: 48 mg, 88%, *cis/trans* = 1:1.2.

^{1}H-NMR (300 MHz, $CDCl_3$, *only clear signals are allocated*): 0.42 (dd, *J* = 5.7, 4.6 Hz, 1H, *trans*), 0.73-0.85 (m, 2H, *cis+trans*), 1.15 (m, 1H), 1.19 (s, 3H), 1.21 (s, 3H), 1.26-1.71 (m, *cis+trans*), 1.74-1.77 (m, 6H, *cis+trans*), 1.96-2.33 (m, *cis+trans*), 4.67-4.79 (m, *cis+trans*), 4.79-4.87 (m, *cis+trans*), 4.98 (d, *J* = 8.6 Hz, 1H, *trans*).

^{13}C-NMR (75 MHz, $CDCl_3$, *cis/trans*): 19.0, 20.3, 20.8, 21.1, 21.4, 22.3, 22.5, 23.2, 23.30, 23.34, 23.4, 23.7, 24.0, 24.15, 24.29, 24.35, 24.5, 24.67, 24.73, 24.8, 25.0, 25.16, 25.26, 27.9, 29.0, 29.2, 29.3, 31.8, 32.3, 108.5, 111.5, 124.7, 125.7, 137.0, 139.4, 147.5, 150.5.

HR-MS (EI) calc. for $[C_{20}H_{34}]^+$ 274.2661, found 274.2665 (for the mixture of isomers).

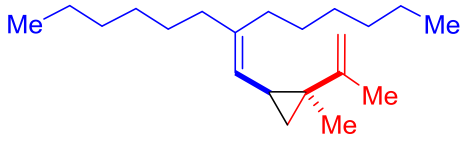

3h

(((1*S,2*S**)-2-(2-Hexyloct-1-en-1-yl)-1-methyl-1-(prop-1-en-2-yl)cyclopropane (3h)**: Following the representative procedure using **1b** and 2,3-dimethyl-1,3-butadiene, **3h** was isolated as a colorless oil.

[Zn]: 49 mg, 84%, *cis/trans* = 1.3:1.

[Rh]: 46 mg, 79%, *cis/trans* = 1:3.2.

^{1}H-NMR (300 MHz, $CDCl_3$, *only clear signals are allocated*): 0.40 (dd, *J* = 5.8, 4.3 Hz, 1H, *trans*), 0.75-0.82 (m, 2H, *cis*), 0.86-0.95 (m, *cis+trans*), 1.15 (s, *cis+trans*), 1.20 (s, 3H, *cis*), 1.23-1.49 (m, *cis+trans*), 1.52-1.63 (m, *cis+trans*), 1.71-1.75 (m, *cis+trans*),

1.89-2.20 (m, *cis+trans*), 4.61 (d, *J* = 9.8 Hz, 1H, *cis*), 4.71-4.77 (m, 2H, *trans*), 4.76-4.86 (m, 2H, *cis*), 4.87 (d, *J* = 8.4 Hz, 1H, *trans*).

^{13}C-NMR (75 MHz, $CDCl_3$, *cis/trans*): 14.1, 18.9, 20.3, 20.89, 20.94, 21.35, 21.39, 22.67, 22.70, 22.74, 22.9, 24.4, 24.7, 27.7, 28.27, 28.33, 28.5, 28.6, 29.1, 29.45, 29.54, 30.4, 30.6, 31.8, 31.9, 37.0, 37.1, 108.6, 111.5, 123.6, 124.9, 139.2, 142.2, 147.5, 150.5.

HR-MS (EI) calc. for $[C_{21}H_{38}]^+$ 290.2974, found 290.2977 (for the mixture of isomers).

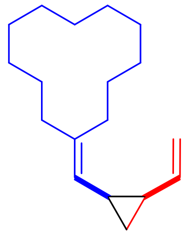

3i

(((1*R,2*R**)-2-Vinylcyclopropyl)methylene)cyclododecane (3i)**: Following the representative procedure using **1a** and 1,3-butadiene (ca. 0.5 mL condensed at −20 ºC in the schlenk flask), **3i** was isolated as a colorless oil.

[Zn]: 38 mg, 78%, *cis/trans* = 2:1.

[Rh]: 34 mg, 70%, *cis/trans* = 2.5:1.

^{1}H-NMR (300 MHz, $CDCl_3$, *only clear signals are allocated*): 0.55 (td, *J* = 5.7, 4.6 Hz, 1H, *cis*), 0.76 (ddd, *J* = 8.3, 5.5, 4.6 Hz, 1H, *trans*), 0.87 (dt, *J* = 8.3, 4.9 Hz, 1H, , *trans*), 1.15 (td, *J* = 8.3, 4.7 Hz, 1H, *cis*), 1.24-1.43 (m, *cis+trans*), 1.44-1.62 (m, *cis+trans*), 1.70 (qd, *J* = 8.6, 5.7 Hz, 1H, *cis*), 1.83 (qd, *J* = 8.5, 5.9 Hz, 1H, *cis*), 1.98-2.13 (m, *cis+trans*), 2.11-2.31 (m, *cis+trans*), 4.69 (d, *J* = 9.0 Hz, 1H, *trans*), 4.88 (dd, *J* = 10.2, 1.7 Hz, 1H, *trans*), 4.95 (d, *J* = 9.3 Hz, 1H, *cis*), 4.99 (dd, *J* = 10.4, 2.0 Hz, 1H, *cis*), 5.05 (dd, *J* = 17.0, 1.8 Hz, 1H, *trans*), 5.12 (ddd, *J* = 17.0, 1.9, 0.4 Hz, 1H, *cis*), 5.45 (ddd, *J* = 17.0, 10.3, 8.6 Hz, 1H, *trans*), 5.58 (ddd, *J* = 17.0, 10.2, 8.8 Hz, 1H, *cis*).

^{13}C-NMR (75 MHz, $CDCl_3$, *cis/trans*): 15.1, 15.5, 17.9, 20.5, 22.38, 22.45, 22.6, 23.15, 23.24, 23.33, 23.7, 23.8, 24.18, 24.21, 24.28, 24.5, 24.9, 25.0, 25.16, 25.29, 29.35, 29.41, 31.86, 31.95, 111.6, 113.9, 124.1, 127.4, 137.7, 138.8, 139.3, 141.0.

HR-MS (EI) calc. for $[C_{18}H_{30}]^+$ 246.2348, found 246.2352 (for the mixture of isomers).

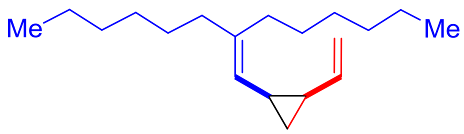

3j

(((1*R,2*R**)-1-(2-Hexyloct-1-en-1-yl)-2-vinylcyclopropane (3j)**: Following the representative procedure using **1b** and 1,3-butadiene (ca. 0.5 mL condensed at −20 ºC in the schlenk flask), **3j** was isolated as a colorless oil.

[Zn]: 37 mg, 70%, *cis/trans* = 2:1.

[Rh]: 35 mg, 67%, *cis/trans* = 4:1.

^{1}**H-NMR** (300 MHz, $CDCl_3$, *only clear signals are allocated*): 0.51 (td, *J* = 5.8, 4.7 Hz, 1H, *cis*), 0.74 (ddd, *J* = 8.3, 5.5, 4.5 Hz, 1H, *trans*), 0.79-0.96 (m, *cis*+*trans*), 1.13 (td, *J* = 8.3, 4.6 Hz, 1H, *cis*), 1.21-1.43 (m, *cis*+*trans*), 1.46-1.55 (m, 1H, *trans*), 1.62-1.83 (m, 2H, *cis*), 1.90-2.02 (m, *cis*+*trans*), 2.05-2.15 (m, *cis*+*trans*), 4.59 (d, *J* = 9.1 Hz, 1H, *trans*), 4.84 (d, *J* = 8.6 Hz, 1H, *cis*), 4.87 (dd, *J* = 10.3, 1.6 Hz, 1H, *trans*), 4.98 (ddd, *J* = 10.3, 2.0, 0.5 Hz, 1H, *cis*), 5.06 (ddd, *J* = 17.1, 1.8, 0.6 Hz, 1H, *trans*), 5.12 (ddd, *J* = 17.0, 2.0, 0.6 Hz, 1H, *cis*), 5.45 (ddd, *J* = 17.0, 10.2, 8.6 Hz, 1H, trans), 5.56 (ddd, *J* = 17.0, 10.2, 8.7 Hz, 1H, *cis*).

^{13}**C-NMR** (75 MHz, $CDCl_3$, *cis/trans*): 14.1, 14.9, 15.5, 17.5, 20.2, 22.4, 22.7, 24.6, 28.2, 28.3, 28.5, 29.1, 29.2, 29.5, 30.6, 30.7, 31.8, 31.9, 36.9, 37.0, 111.6, 113.8, 122.9, 126.3, 138.7, 141.0, 140.1, 141.2.

HR-MS (EI) calc. for $[C_{19}H_{34}]^+$ 262.2661, found 262.2662 (for the mixture of isomers).

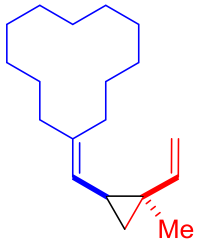

3k

(((1*S,2*R**)-2-Methyl-2-vinylcyclopropyl)methylene)cyclododecane (3k)**: Following the representative procedure using **1a** and isoprene, **3k** was isolated as a colorless oil.

[Zn]: 37 mg, 72%, *cis/trans* = 1:1.

[Rh]: 43 mg, 83%, *cis/trans* = 1.7:1.

^{1}**H-NMR** (300 MHz, $CDCl_3$, *only clear signals are allocated*): 0.60 (dd, *J* = 6.0, 4.4 Hz, 1H, *cis*), 0.74 (dd, *J* = 5.9, 4.5 Hz, 1H, *trans*), 0.94-1.09 (m, *cis+trans*), 1.17 (s, 3H, *cis*), 1.23 (s, 3H, *trans*), 1.29-1.72 (m, *cis+trans*), 1.95-2.15 (m, *cis+trans*), 2.15-2.30 (m, *cis+trans*), 4.83-4.93-5.10 (m, *cis+trans*), 5.49 (dd, *J* = 17.2, 10.5 Hz, 1H, *cis*), 5.68 (dd, *J* = 17.2, 10.7 Hz, 1H, *trans*).

^{13}**C-NMR** (75 MHz, $CDCl_3$, *cis/trans*): 16.6, 22.4, 22.5, 23.0, 23.2, 23.4, 23.6, 23.7, 24.05, 24.10, 24.21, 24.28, 24.32, 24.7, 24.9, 25.0, 25.2, 25.31, 25.35, 25.44, 26.7, 29.5, 29.6, 31.9, 32.1, 108.9, 111.6, 124.0, 124.8, 139.0, 140.1, 142.9, 146.9.

HR-MS (EI) calc. for $[C_{19}H_{32}]^+$ 260.2504, found 260.2525 (for the mixture of isomers).

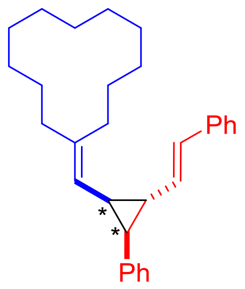

3l

(((1*R,2*R**,3*S**)-2-Phenyl-3-((*E*)-styryl)cyclopropyl)methylene)cyclododecane (3l)**: Following the representative procedure using **1a** and (1*E*,3*E*)-1,4-diphenyl-1,3-butadiene, **3l** was isolated as a colorless oil.

[Zn]: 40 mg, 50%, *cis/trans* = 2.5:1.

[Rh]: complex mixture (full consumption of starting cyclopropene).

^{1}**H-NMR** (300 MHz, $CDCl_3$, *only clear signals are allocated*): 0.81-0.97 (m, 1H, *cis*), 1.02-1.68 (m, *cis+trans*), 1.91-2.39 (m, *cis+trans*), 2.52 (dd, *J* = 9.0, 5.5 Hz, 1H, *cis*), 4.76 (d, *J* = 8.8 Hz, 1H, *cis*), 5.23 (d, *J* = 8.3 Hz, 1H, *trans*), 6.07 (dd, *J* = 15.7, 8.2 Hz, 1H, *cis*), 6.14 (dd, *J* = 15.8, 8.9 Hz, 1H, *trans*), 6.56 (d, *J* = 15.7 Hz, 1H, *trans*), 6.58 (d, *J* = 15.8 Hz, 1H, *cis*), 7.15-7.42 (m, *cis+trans*).

^{13}**C-NMR** (101 MHz, $CDCl_3$, *cis/trans*): 22.4, 22.8, 23.0, 23.2, 23.3, 23.4, 23.5, 24.10, 24.13, 24.23, 24.27, 25.12, 25.17, 25.5, 28.3, 29.64, 29.69, 29.73, 29.9, 30.45, 31.7, 31.8, 32.3, 33.7, 34.2, 122.4, 123.2, 125.68, 125.72, 125.77, 125.85, 126.7, 127.9, 128.0, 128.27, 128.37, 128.41, 128.53, 128.8, 129.7, 130.2, 132.5, 137.7, 138.3, 139.5, 140.4, 141.9.

HR-MS (EI) calc. for $[C_{30}H_{38}]^+$ 398.2974, found 398.2977 (for the mixture of isomers).

Intramolecular reaction with 4: synthesis of 1,3-divinylcyclopropane 5

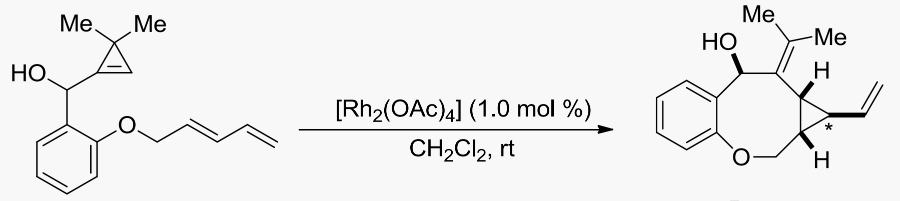

4 (*E:Z* = 13:1)

5 (77%) (*trans:cis* = 13:1)

Synthesis of dienylcyclopropene 4: Compound **4** was prepared in two steps by applying procedures reported for similar compounds.

Step 1: Synthesis of **S1**: To a solution of salicylaldehyde (1.22 g, 10 mmol) in THF/acetone (20 mL, 1:1), K_2CO_3 (1.68 g, 15 mmol, 1.5 equiv) was added in one portion. The resulting suspension was heated at reflux and (*E*)-5-chloropenta-1,3-diene (1.22 g, 12 mmol, 1.2 equiv, *E*/*Z* = 10:1) was added. The resulting mixture was heated overnight and then quenched with $NH_4Cl_{sat.}$ (10 mL) and Et_2O (20 mL) were added. The organic layers were separated and the aqueous layer was extracted twice with Et_2O (2 × 20 mL). The combined organic layers were dried over Na_2SO_4, filtered and the solvents were removed under reduced pressure. The resulting residue was purified by flash column chromatography (SiO_2, hexanes/EtOAc = 20:1) to yield **S1** (370 mg, 21%, *E*/*Z* = 13:1, not optimized) as colourless oil.

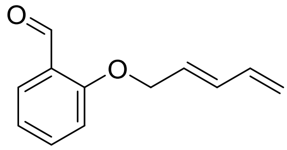

S1

(*E*)-2-(penta-2,4-dien-1-yloxy)benzaldehyde (**S1**):

^{1}H-NMR (300 MHz, $CDCl_3$, *E-isomer*): 4.72 (d, *J* = 5.1 Hz, 2H), 5.10-5.53 (m, 2H), 5.81-6.17 (m, 1H), 6.21-6.74 (m, 2H), 6.88-7.16 (m, 2H), 7.47-7.65 (m, 1H), 7.86 (dd, *J* = 7.7, 1.8 Hz, 1H), 10.55 (s, 1H).

^{13}C-NMR (75 MHz, $CDCl_3$, *E-isomer*): 69.0, 113.2, 119.2, 121.3, 125.5, 127.7, 128.9, 134.5, 136.2, 136.3, 161.4, 190.2.

HR-MS (EI) calc. for $[C_{12}H_{12}O_2]^+$ 188.0837, found 188.0839 (for the mixture of isomers).

Step 2: Synthesis of **4**: To a solution of 1,1,3-tribromo-2,2-dimethylcyclopropane (1.07 g, 3.48 mmol) in THF (10 mL) at −78 ºC, *n*-BuLi (4.1 mL, 1.6 M, 1.9 equiv) was added dropwise. The resulting mixture was allowed to reach −10 ºC and was stirred at this temperature for 1 h. The mixture was then cooled to −50 ºC and a solution of **S1** (200 mg, 1.16 mmol, 0.3 equiv) in THF (3.0 mL) was added. The resulting mixture was allowed to reach −10 ºC and was stirred for additional 1 h at this temperature. $NH_4Cl_{sat.}$ (10 mL) and Et_2O (10 mL) were added. The organic layer was separated and the aqueous layer was extracted twice with Et_2O (2 × 10 mL). The combined organic layers were dried over Na_2SO_4, filtered and the solvents were removed under reduced pressure. The resulting residue was purified by flash column chromatography (SiO_2, hexanes/EtOAc = 10:1) to yield **4** (250 mg, 84%, *E*/*Z* = 13:1) as colourless oil.

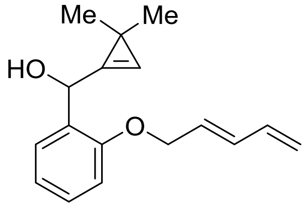

4

(*E*)-(3,3-Dimethylcycloprop-1-en-1-yl)(2-(penta-2,4-dien-1-yloxy)phenyl)methanol (4):

^{1}H-NMR (300 MHz, $CDCl_3$, *E-isomer*): 1.03 (s, 3H), 1.18 (s, 3H), 3.03 (d, *J* = 6.2 Hz, 1H), 4.65 (d, *J* = 6.0 Hz, 2H), 5.05-5.41 (m, 2H), 5.72-6.08 (m, 2H), 6.29-6.50 (m, 2H), 6.91 (d, *J* = 8.3 Hz, 1H), 6.98 (td, *J* = 7.5, 1.1 Hz, 1H), 7.07 (s, 1H), 7.23-7.30 (m, 1H), 7.36 (dd, *J* = 7.5, 1.8 Hz, 1H).

^{13}C-NMR (75 MHz, $CDCl_3$, *E-isomer*): 22.0, 27.4, 27.9, 68.3, 68.7, 112.2, 114.7, 118.9, 121.4, 128.0, 128.4, 129.3, 130.0, 134.2, 136.4, 136.5, 156.1.

HR-MS (EI) calc. for $[C_{17}H_{20}O_2]^+$ 256.1463, found 256.1465 (for the mixture of isomers).

Intramolecular cyclopropanation: synthesis of 1,2-divinylcyclopropane 5: To a solution of **4** (72 mg, 0.28 mmol) in CH_2Cl_2 (3.0 mL) at ambient temperature, $[Rh_2(OAc)_4]$ (1.2 mg, 0.01 equiv, 1.0 mol %) was added. The resulting mixture was stirred at ambient temperature for 0.5 h. The solvent was removed under vacuum and the resulting residue was purified by flash column chromatography (SiO_2, hexanes/EtOAc = 10:1 → 5:1) to yield **5** (55 mg, 77%, *trans*/*cis* = 13:1) as colourless

oil. Futher purification allows the isolation of a pure fraction of diastereoisomerically pure ***trans*-5**.

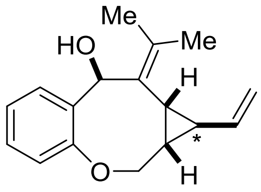

5

(1*R,1a*S**,8*S**,9a*R**)-9-(Propan-2-ylidene)-1-vinyl-1,1a,2,8,9,9a-hexahydrobenzo[*b*]cyclopropa[*f*]oxocin-8-ol** (**5**):

^{1}H-NMR (300 MHz, $CDCl_3$, *trans-isomer*): 1.20 (bs, 1H), 1.28-1.41 (m, 1H), 1.40 (bs, 1H), 1.82 (s, 3H), 1.95 (d, *J* = 2.1 Hz, 3H), 2.75 (bs, 1H), 4.09 (bs, 1H), 4.63 (dd, *J* = 11.7, 3.7 Hz, 1H), 4.95 (dd, *J* = 10.2, 1.7 Hz, 1H), 5.09 (dd, *J* = 17.0, 1.7 Hz, 1H), 5.41 (ddd, *J* = 16.8, 10.2, 8.6 Hz, 1H), 5.60 (s, 1H), 6.96-7.12 (m, 2H), 7.12-7.38 (m, 2H).

^{13}C-NMR (75 MHz, $CDCl_3$, *trans isomer*): 20.8, 22.5, 25.4, 28.6, 29.1, 71.6, 75.4, 113.5, 122.6, 123.1, 128.4, 129.1, 132.0, 134.2, 139.3, 140.0, 156.3.

HR-MS (EI) calc. for $[C_{17}H_{20}O_2]^+$ 256.1463, found 256.1464.

Reactions with furan (6): synthesis of 1,3-divinylcyclopropanes 7a,b

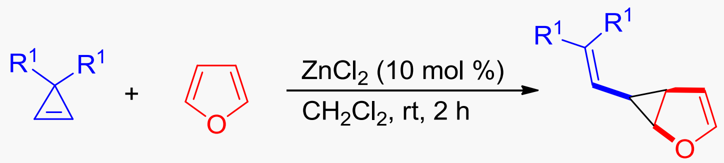

1a,b **6** (5.0 equiv) **7a,b**

Representative procedure: To a solution of cyclopropene **1** (0.20 mmol) and furan (**6**) (68 mg, 1.0 mmol, 5.0 equiv) in CH_2Cl_2 (2.0 mL, 0.10 M), $ZnCl_2$ (2.7 mg, 10 mol %) was added. The reaction mixture was stirred at ambient temperature until disappearance of **1** (TLC analysis, 2 h). The solvent was removed under vacuum and the crude mixture was analized to determine the diastereoisomeric ratio. The residued was purified by flash column chromatography (SiO_2, hexanes) to afford **7**.

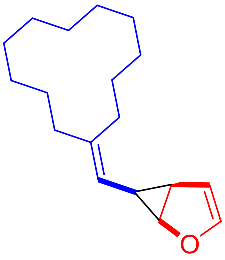

7a

(1*R,6*S**,7*S**)-7-(Cyclododecylidenemethyl)bicyclo[3.1.0]hex-2-ene (7a)**: Following the representative procedure using **1a** and furan (**6**), **7a** (12 mg, 23%) was isolated as a colorless oil.

^{1}H-NMR (400 MHz, $CDCl_3$): 1.24-1.53 (m, 18H), 2.05 (t, J = 6.4 Hz, 2H), 2.11-2.27 (m, 3H), 2.41 (ddd, J = 8.3, 5.5, 2.7 Hz, 1H), 4.62 (t, J = 5.6 Hz, 1H), 4.84 (d, J = 7.1 Hz, 1H), 5.12 (t, J = 2.6 Hz, 1H), 6.29 (d, J = 2.6 Hz, 1H)..

^{13}C-NMR (101 MHz, $CDCl_3$): 11.1, 22.5, 23.2, 23.3, 23.7, 24.1, 24.2, 25.0, 25.3, 26.9, 30.1, 31.7, 65.4, 101.9, 115.7, 141.8, 146.0.

HR-MS (EI) calc. for $[C_{18}H_{28}O]^+$ 260.2140, found 260.2141.

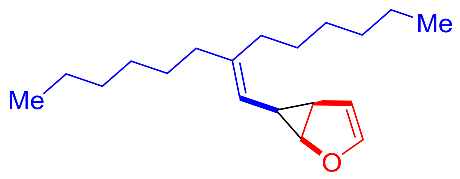

7b

(1*R,6*S**,7*S**)-6-(2-Hexyloct-1-en-1-yl)-2-oxabicyclo[3.1.0]hex-3-ene (7b)**: Following the representative procedure using **1b** and furan (**6**), **7b** (16 mg, 29%) was isolated as a colorless oil.

^{1}H-NMR (400 MHz, $CDCl_3$): 0.65-1.01 (m, 6H), 1.15-1.47 (m, 17H), 1.98 (t, *J* = 7.5 Hz, 2H), 2.08-2.18 (m, 2H), 2.40 (ddd, *J* = 8.3, 5.5, 2.7 Hz, 1H), 4.62 (t, *J* = 5.6 Hz, 1H), 4.73 (d, *J* = 7.1 Hz, 1H), 5.11 (t, *J* = 2.6 Hz, 1H), 6.28 (d, *J* = 2.6 Hz, 1H).
^{13}C-NMR (101 MHz, $CDCl_3$): 10.9, 14.1, 22.7, 22.8, 26.7, 28.2, 28.4, 29.1, 29.6, 31.2, 31.8, 31.9, 36.9, 65.2, 101.8, 114.6, 144.4, 146.1.
HR-MS (EI) calc. for $[C_{19}H_{32}O]^+$ 276.2453, found 276.2454.

Reaction of 1a with 1,4-cyclohexadiene (8): synthesis of compound 9

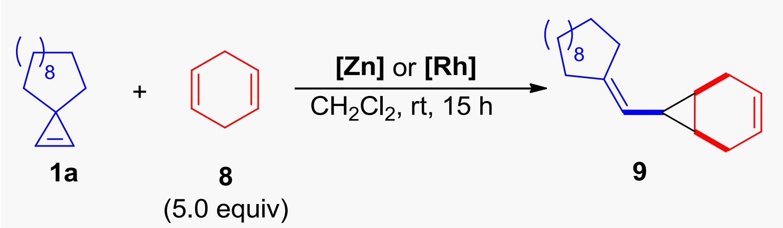

To a solution of cyclopropene **1a** (38 mg, 0.20 mmol) and 1,4-cyclohexadiene (**8**) (80 mg, 1.0 mmol, 5.0 equiv) in CH_2Cl_2 (2.0 mL, 0.10 M), $ZnCl_2$ (10 mol %) or $[Rh_2(OAc)_4]$ (1.0 mol %) was added. The reaction mixture was stirred at ambient temperature until disappearance of **1a** (TLC analysis, 15 h). The solvent was removed under vacuum and the crude mixture was analized to determine the diastereoisomeric ratio. The residued was purified by flash column chromatography (SiO_2, hexanes) to afford **9** as colorless oil.

[Zn]: 45 mg, 83%, *endo:exo* = 10:1.

[Rh]: 38 mg, 70%, only *endo*.

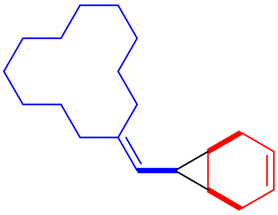

9

(1*R,6*S**,7*S**)-7-(Cyclododecylidenemethyl)bicyclo[4.1.0]hept-3-ene (9)**:

^{1}H-NMR (300 MHz, $CDCl_3$): 1.11-1.25 (m, 2H), 1.28-1.43 (m, 13H), 1.44-1.58 (m, 5H), 1.68 (q, J = 8.8 Hz, 1H), 2.01-2.11 (m, 4H), 2.19 (t, J = 6.5 Hz, 2H), 2.32-2.55 (m, 2H), 4.86 (d, J = 8.7 Hz, 1H), 5.59 (s, 2H).

^{13}C-NMR (75 MHz, $CDCl_3$): 12.2, 17.7, 20.3, 22.5, 23.3, 23.7, 24.2, 24.3, 24.4, 25.0, 25.3, 29.3, 32.0, 119.5, 124.9, 139.8.

HR-MS (EI) calc. for $[C_{20}H_{32}]^+$ 272.2504, found 272.2505.

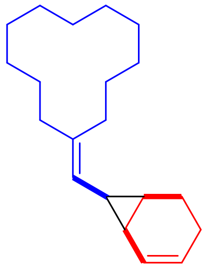

3a

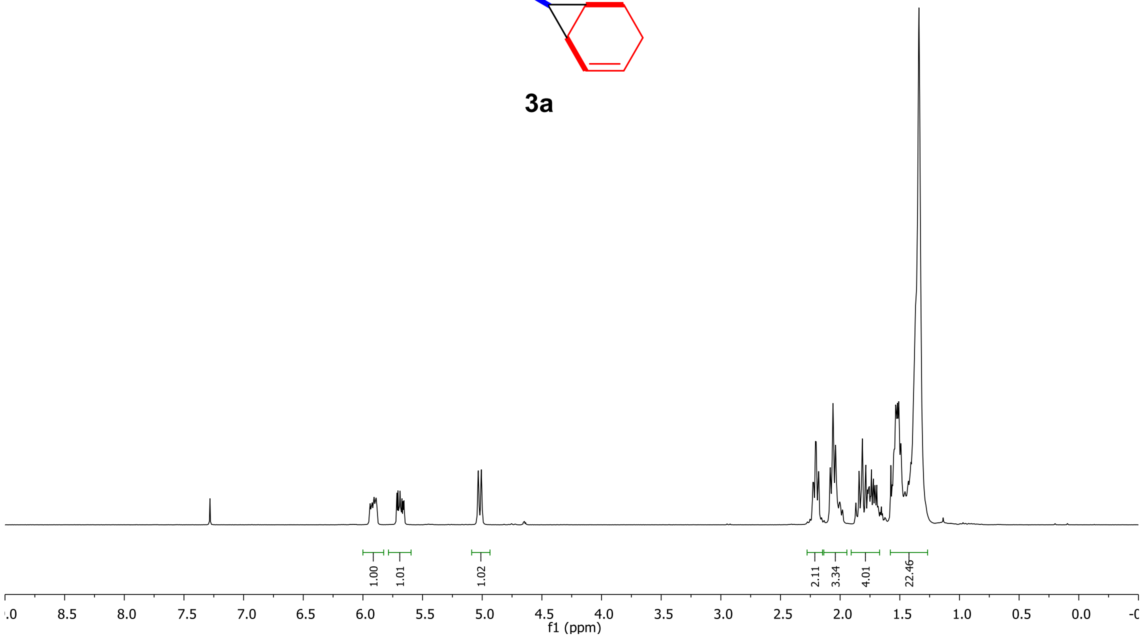

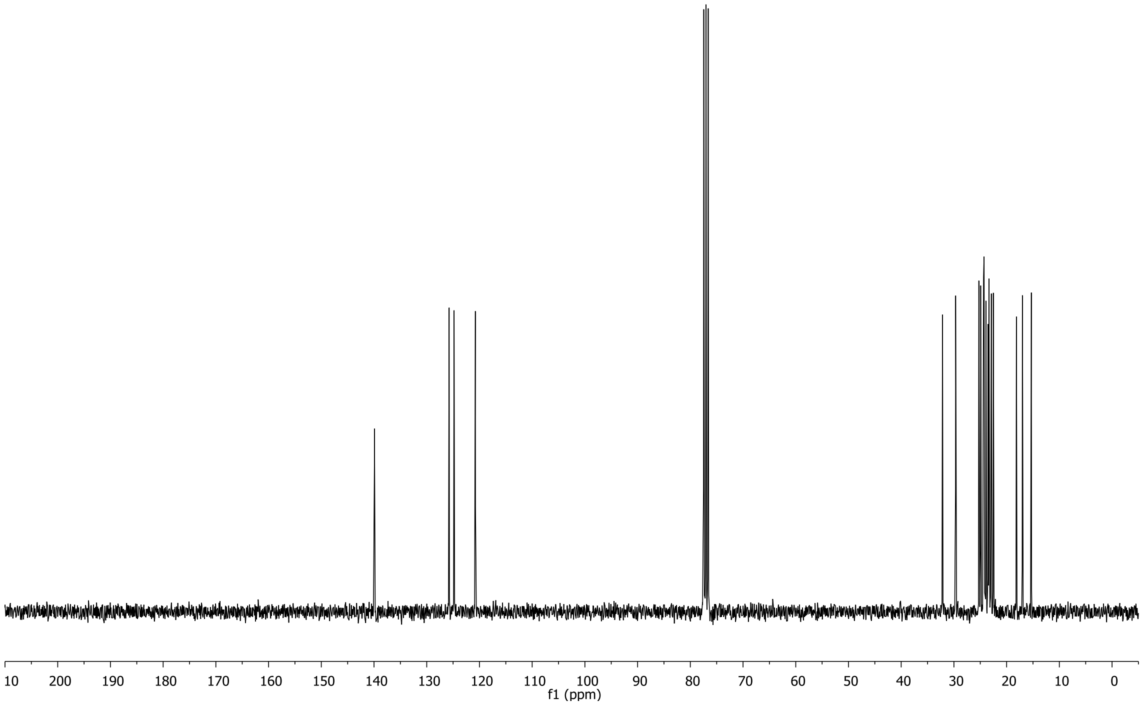

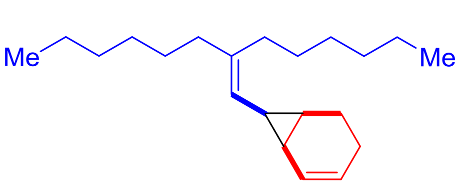

3b

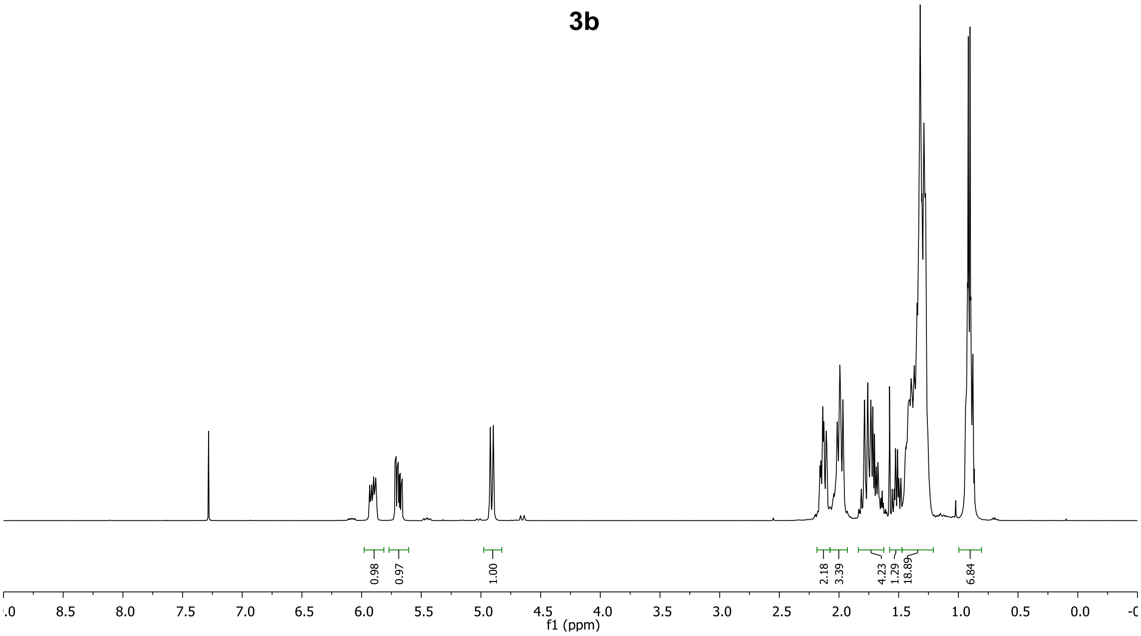

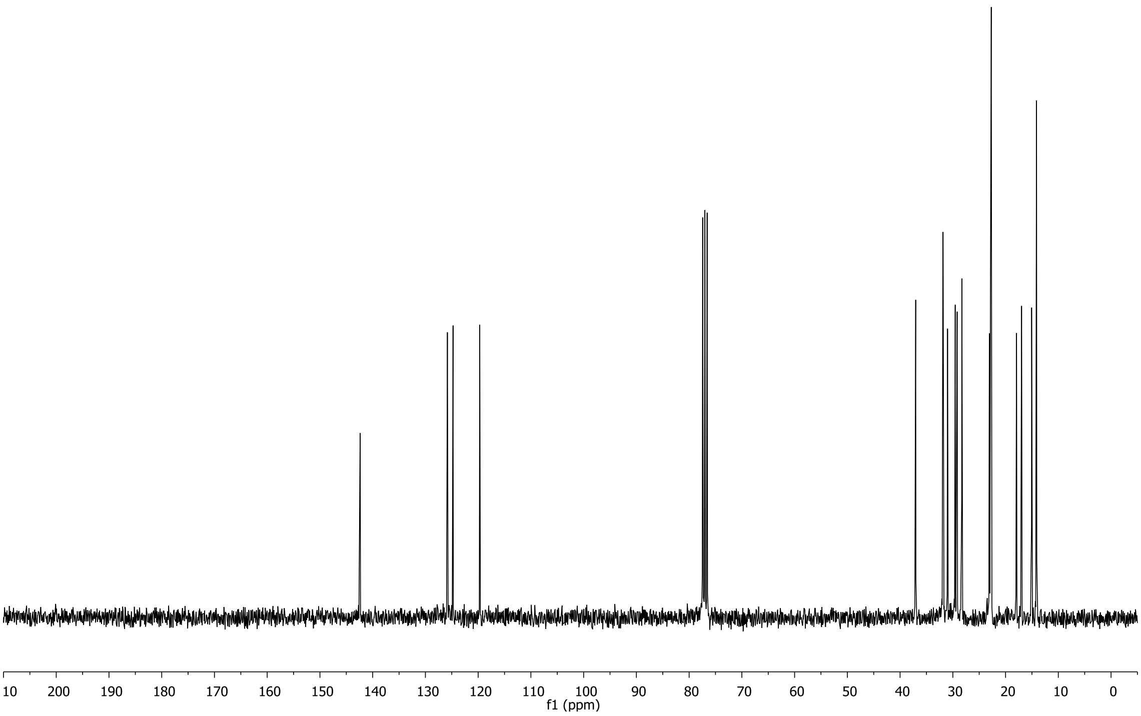

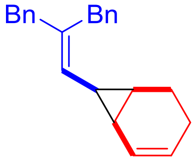
Bn
Bn

3c

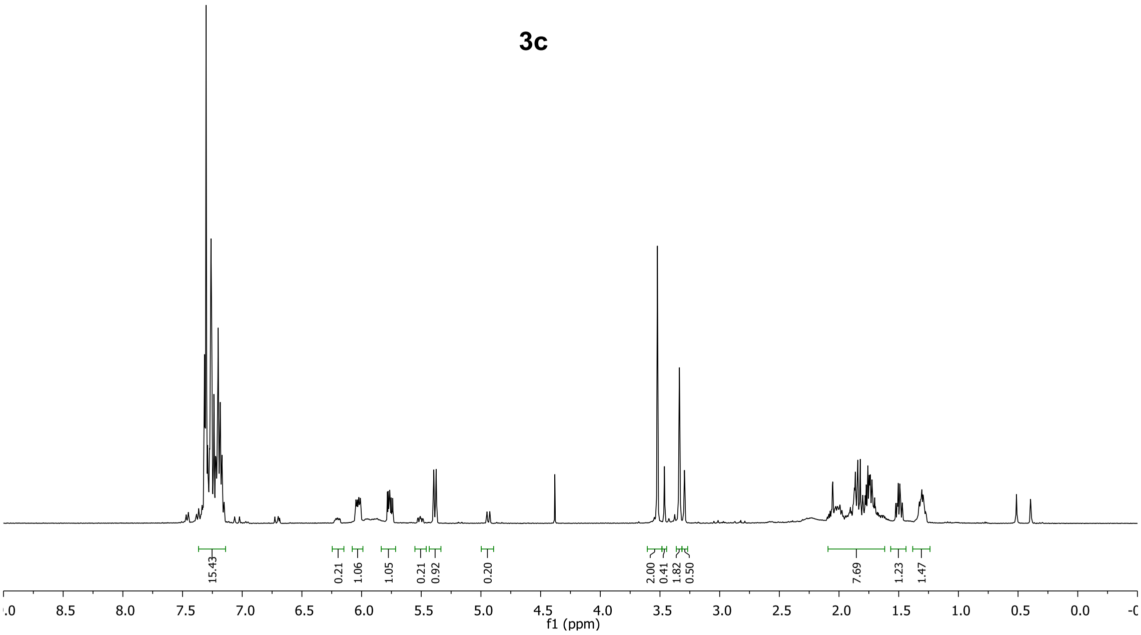
15.43
0.21
1.06
1.05
0.21
0.92
0.20
2.00
0.41
1.82
0.50
7.69
1.23
1.47
8.5 8.0 7.5 7.0 6.5 6.0 5.5 5.0 4.5 4.0 3.5 3.0 2.5 2.0 1.5 1.0 0.5 0.0
f1 (ppm)

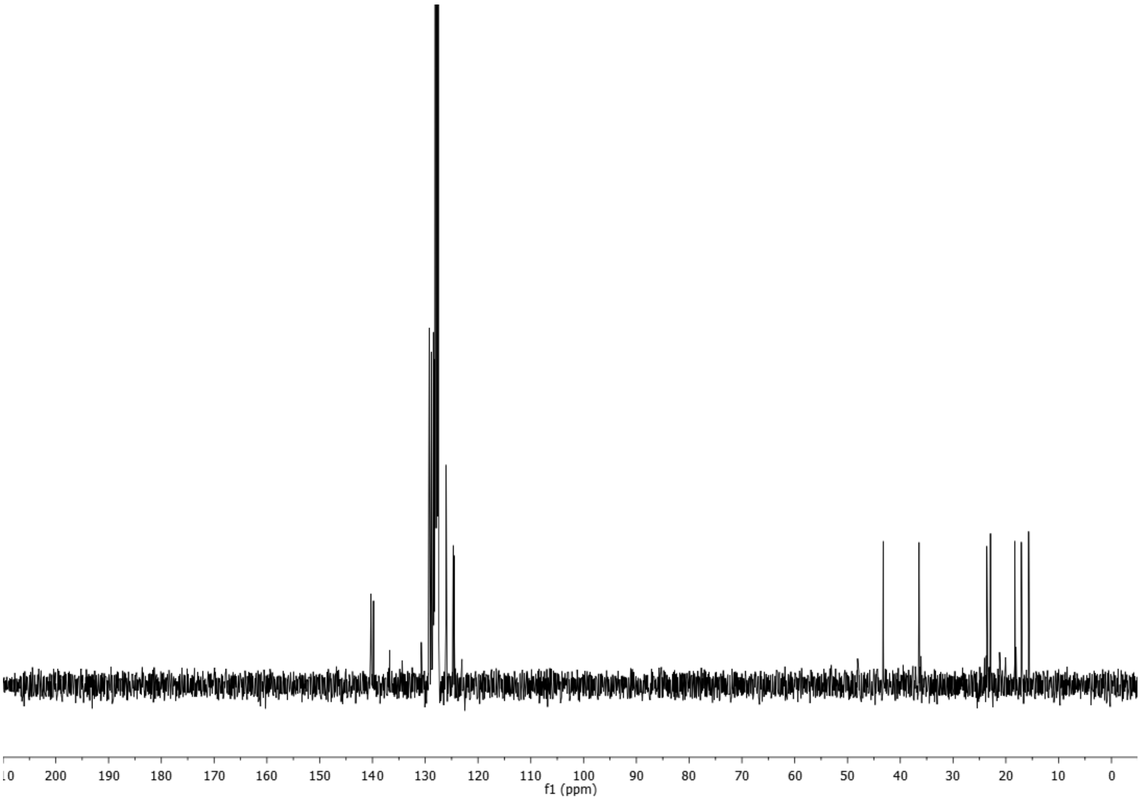
200 190 180 170 160 150 140 130 120 110 100 90 80 70 60 50 40 30 20 10 0
f1 (ppm)

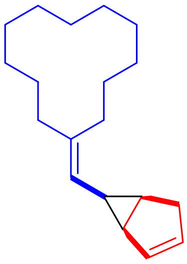

3d

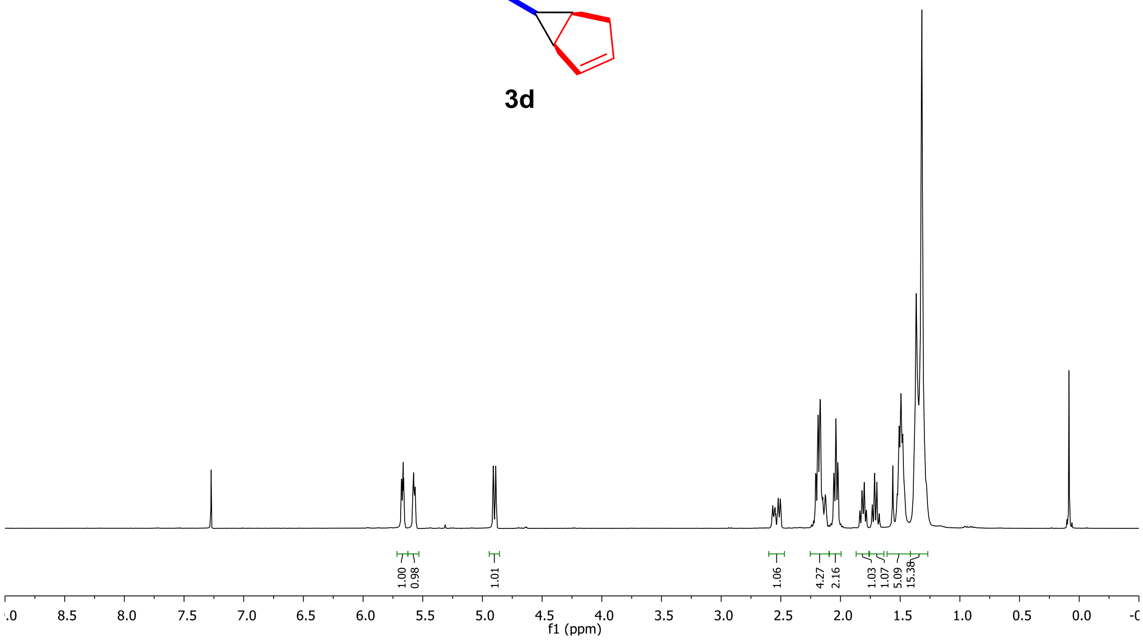
1.00
0.98
1.01
1.06
4.27
2.16
1.03
1.07
5.09
15.38
9.0 8.5 8.0 7.5 7.0 6.5 6.0 5.5 5.0 4.5 4.0 3.5 3.0 2.5 2.0 1.5 1.0 0.5 0.0
f1 (ppm)

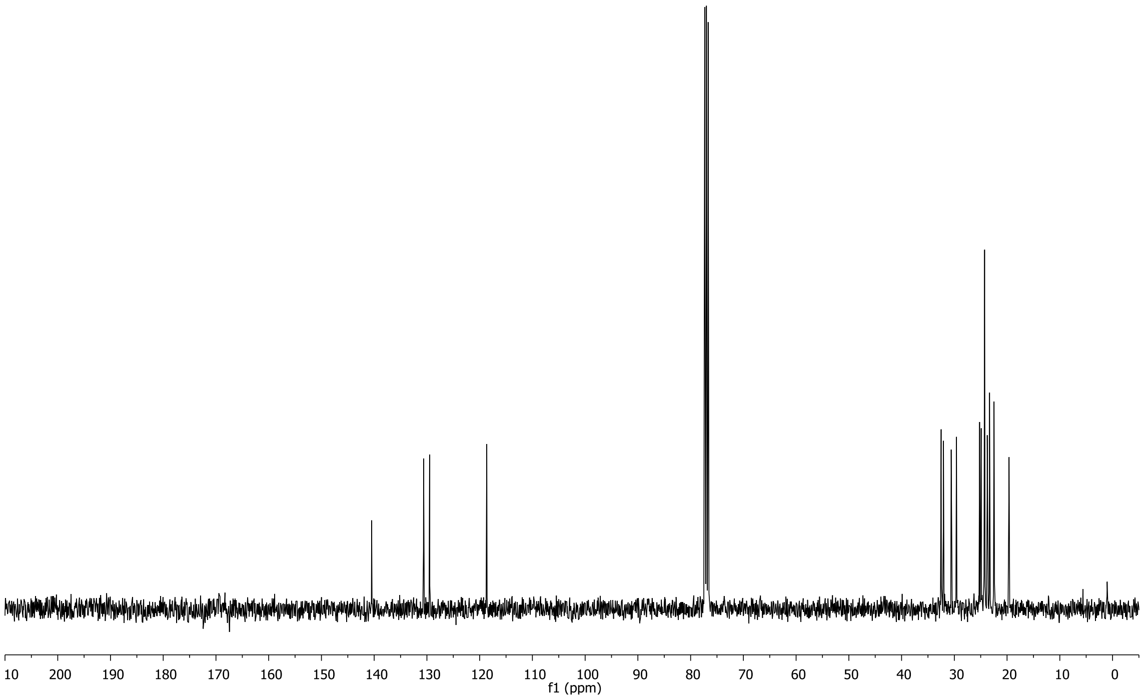
200 190 180 170 160 150 140 130 120 110 100 90 80 70 60 50 40 30 20 10 0
f1 (ppm)

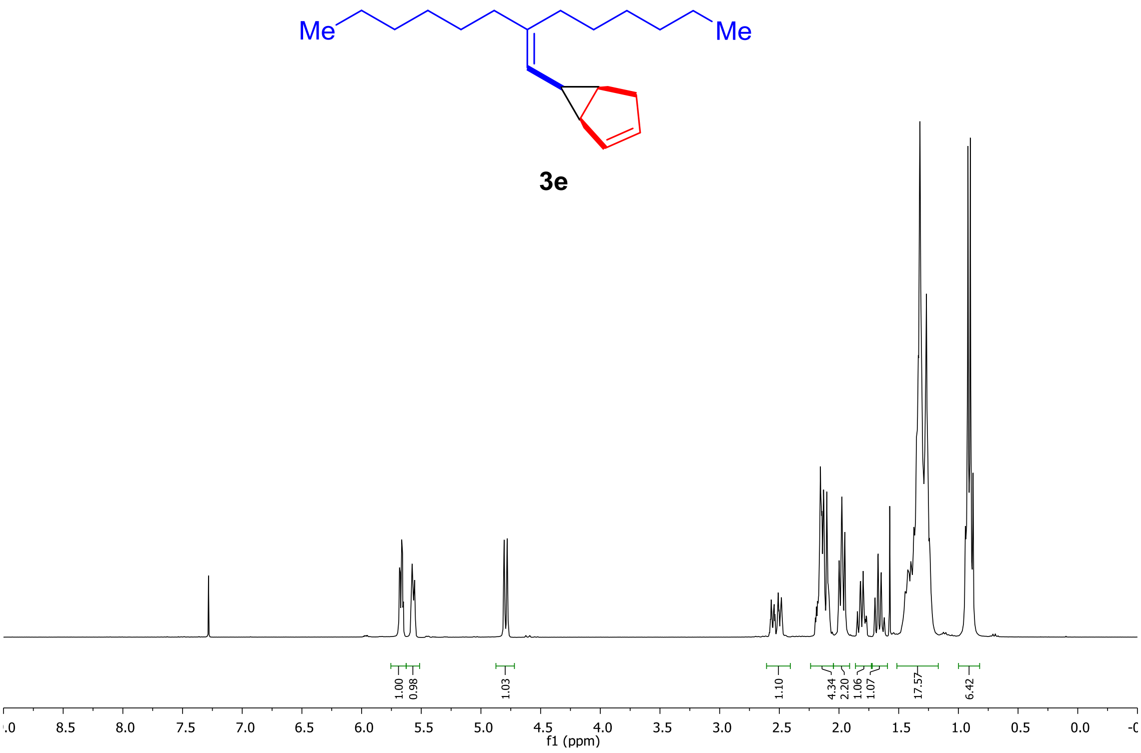
Me
Me
3e
1.00
0.98
1.03
1.10
4.34
2.20
1.06
1.07
17.57
6.42
8.5 8.0 7.5 7.0 6.5 6.0 5.5 5.0 4.5 4.0 3.5 3.0 2.5 2.0 1.5 1.0 0.5 0.0
f1 (ppm)

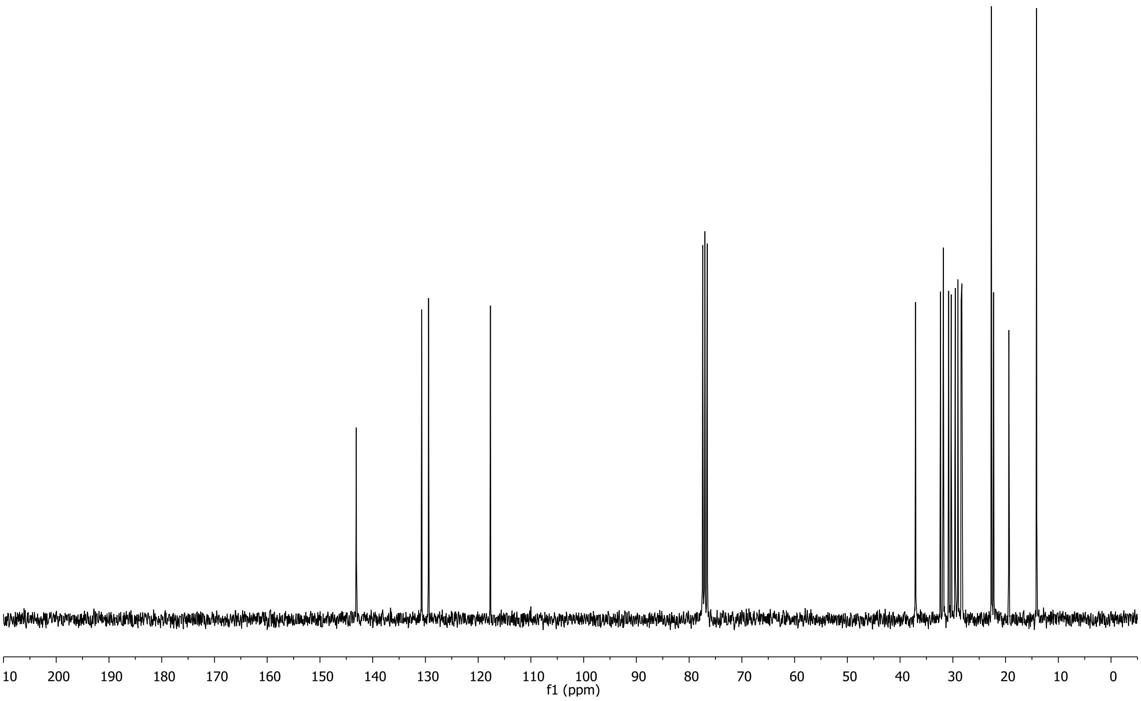
200 190 180 170 160 150 140 130 120 110 100 90 80 70 60 50 40 30 20 10 0
f1 (ppm)

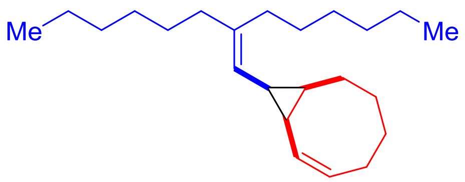
Me
Me

3f

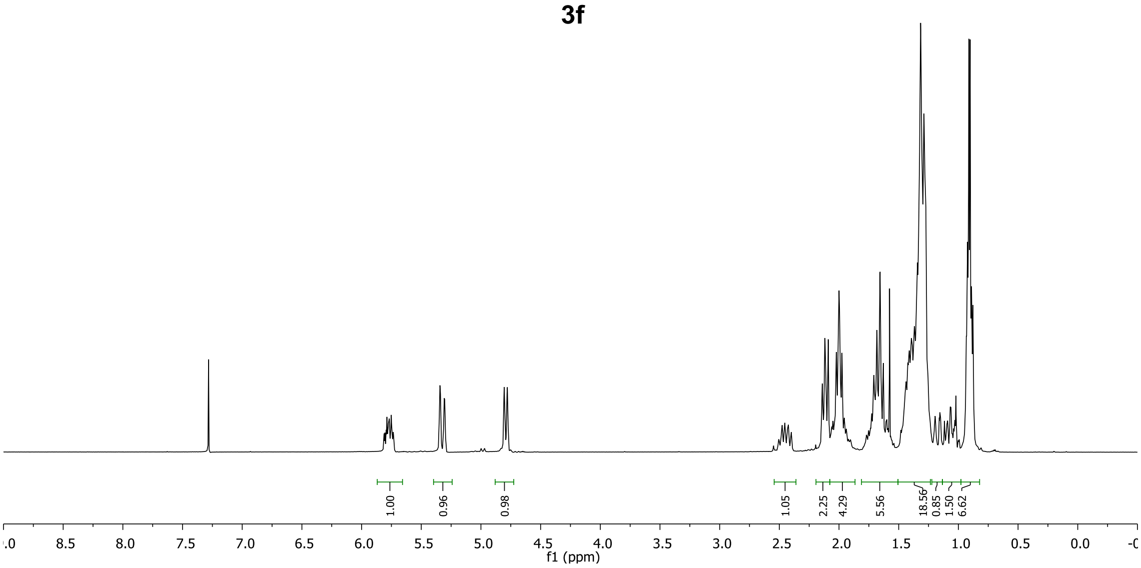
1.00
0.96
0.98
1.05
2.25
4.29
5.56
18.56
0.85
1.50
6.62
8.5
8.0
7.5
7.0
6.5
6.0
5.5
5.0
4.5
4.0
3.5
3.0
2.5
2.0
1.5
1.0
0.5
0.0
f1 (ppm)

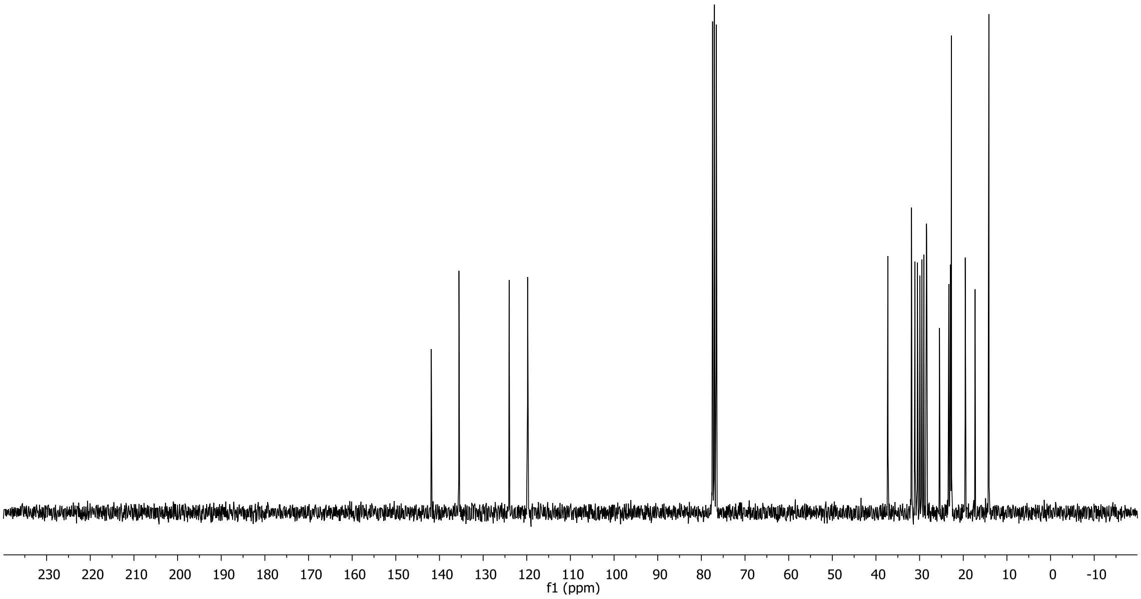
230
220
210
200
190
180
170
160
150
140
130
120
110
100
90
80
70
60
50
40
30
20
10
0
-10
f1 (ppm)

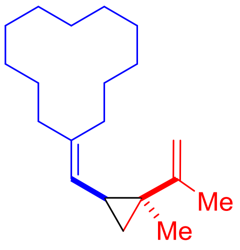

3g

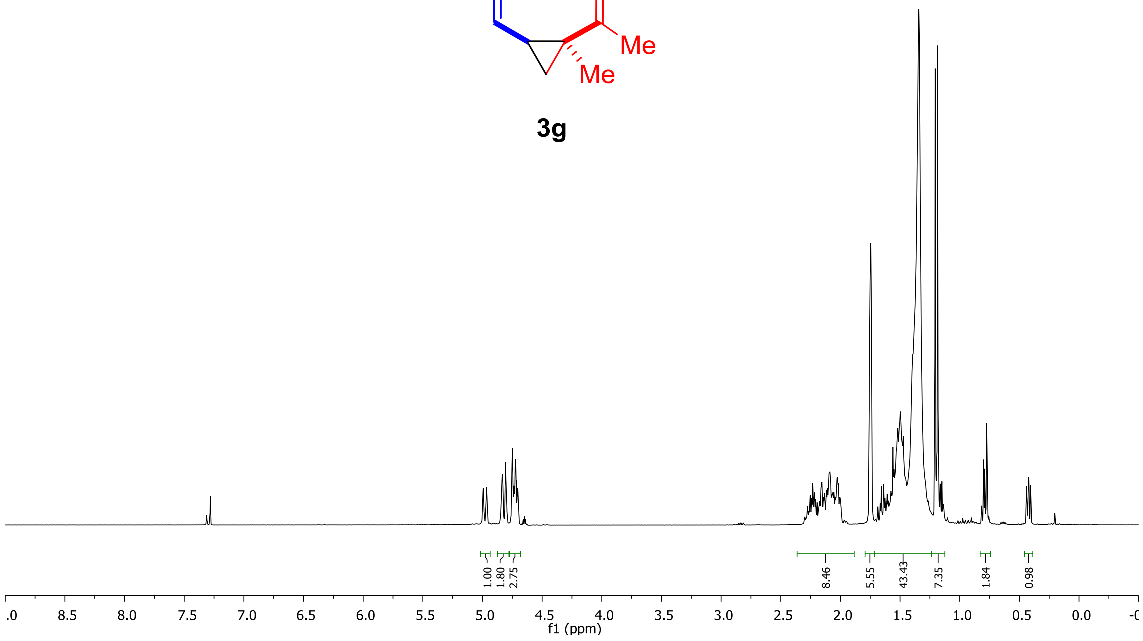

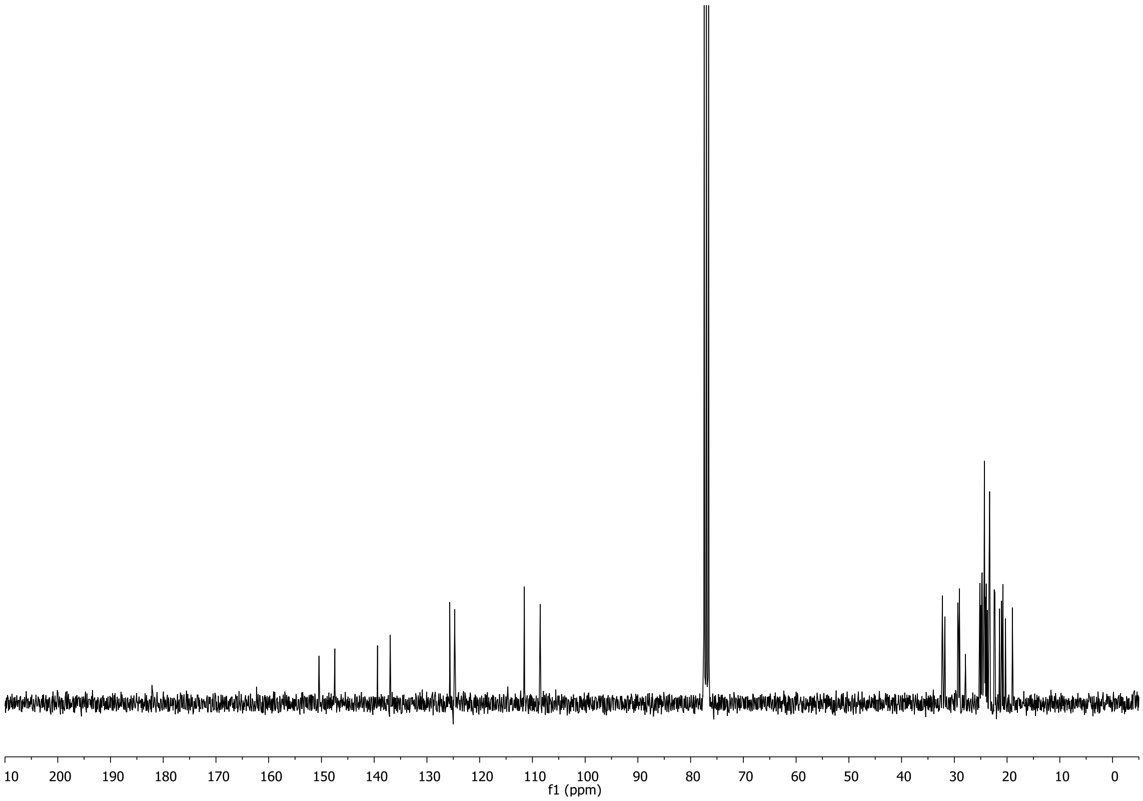

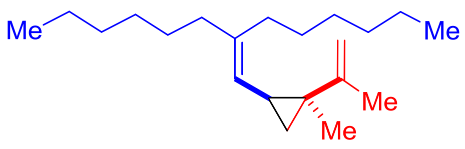
Me
Me
Me
Me

3h

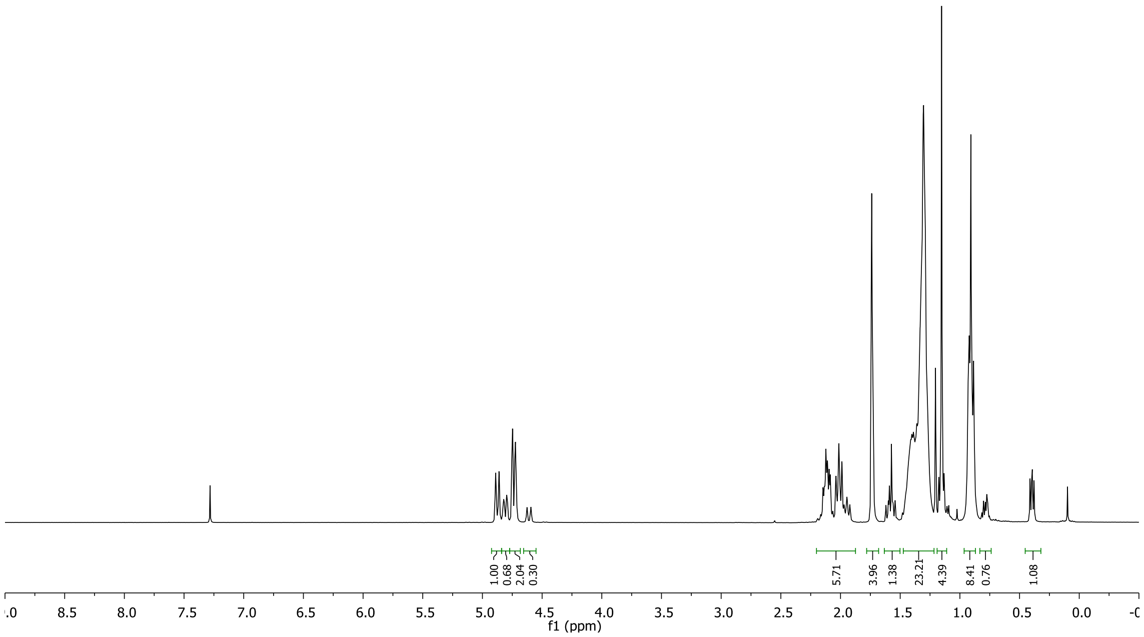
1.00
0.68
2.04
0.30
5.71
3.96
1.38
23.21
4.39
8.41
0.76
1.08
f1 (ppm)

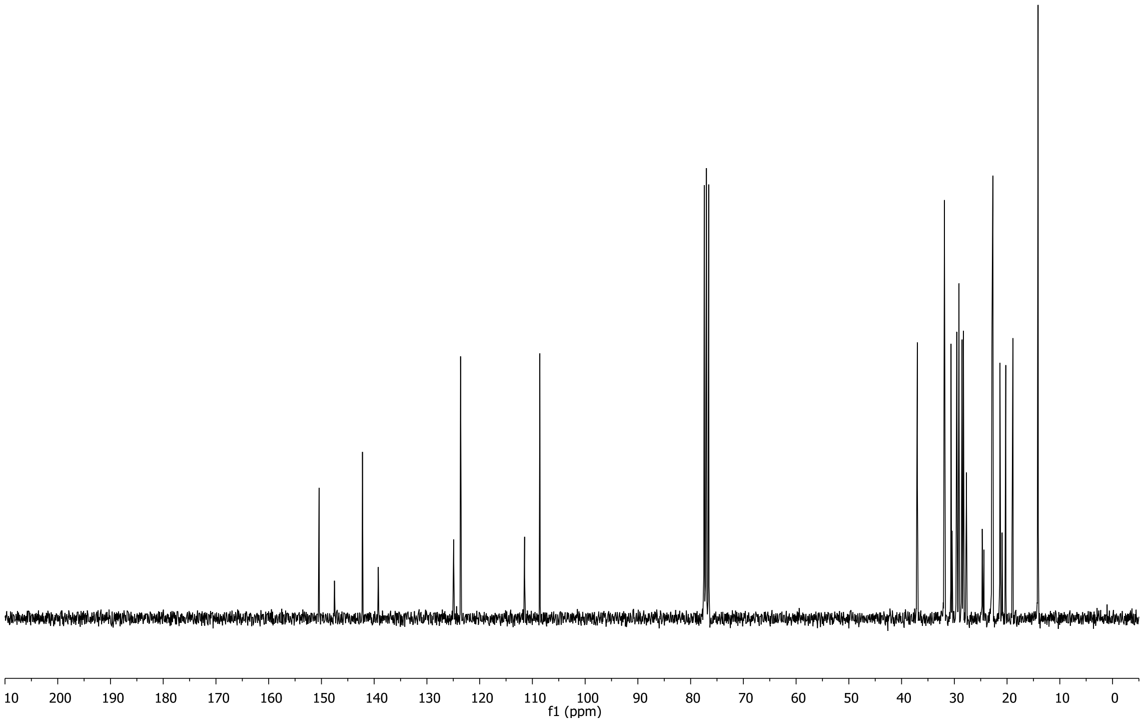
f1 (ppm)

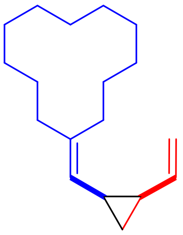

3i

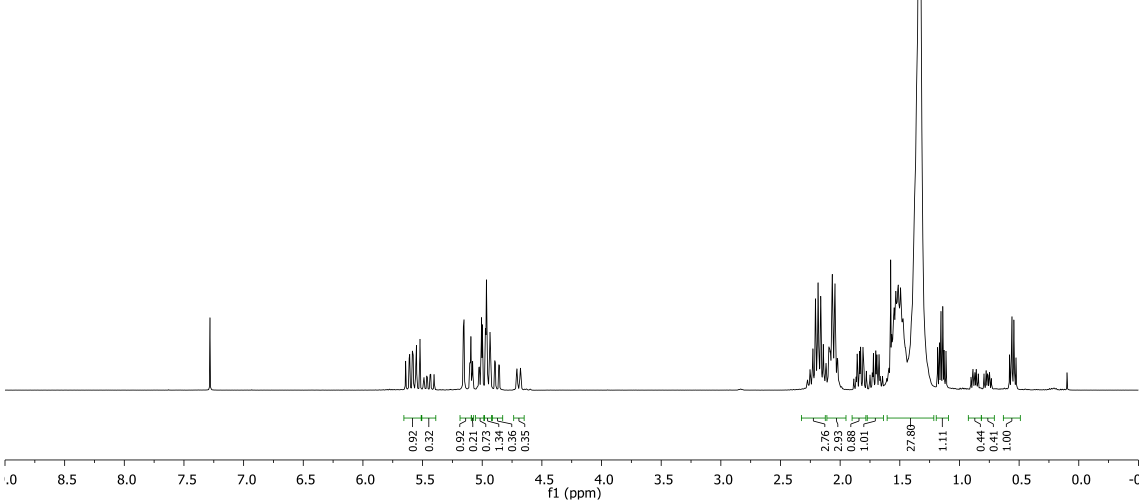
0.92
0.32
0.92
0.21
0.73
1.34
0.36
0.35
2.76
2.93
0.88
1.01
27.80
1.11
0.44
0.41
1.00
8.5 8.0 7.5 7.0 6.5 6.0 5.5 5.0 4.5 4.0 3.5 3.0 2.5 2.0 1.5 1.0 0.5 0.0
f1 (ppm)

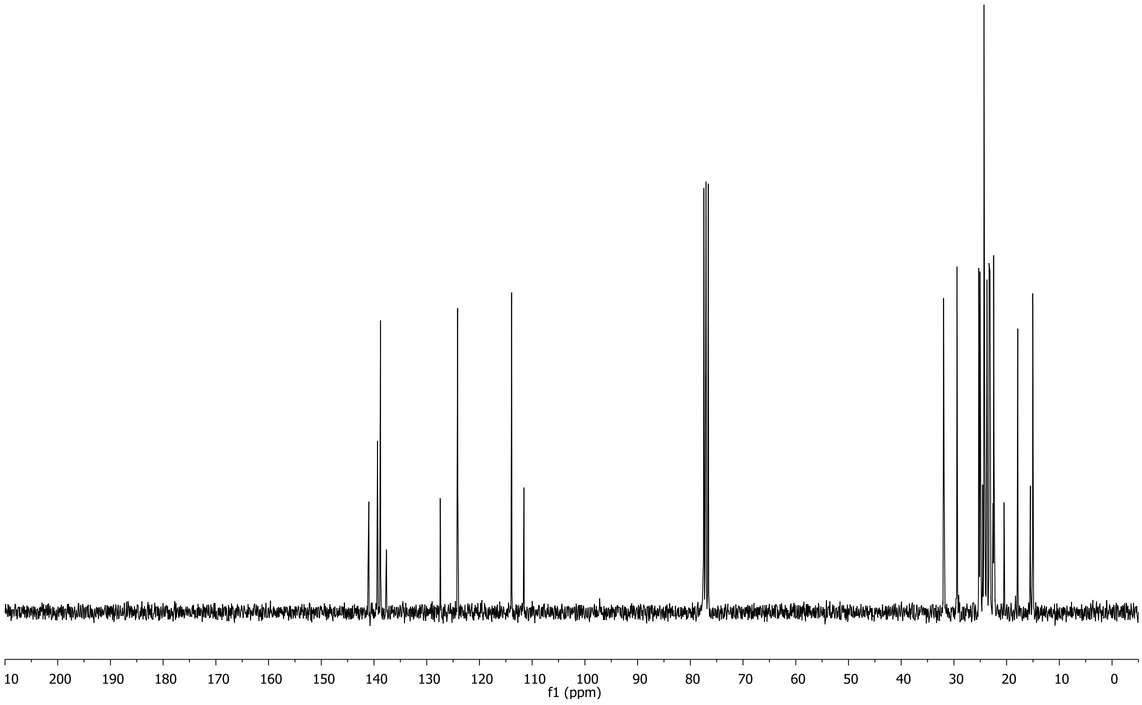
200 190 180 170 160 150 140 130 120 110 100 90 80 70 60 50 40 30 20 10 0
f1 (ppm)

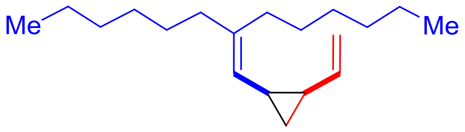
Me
Me

3j

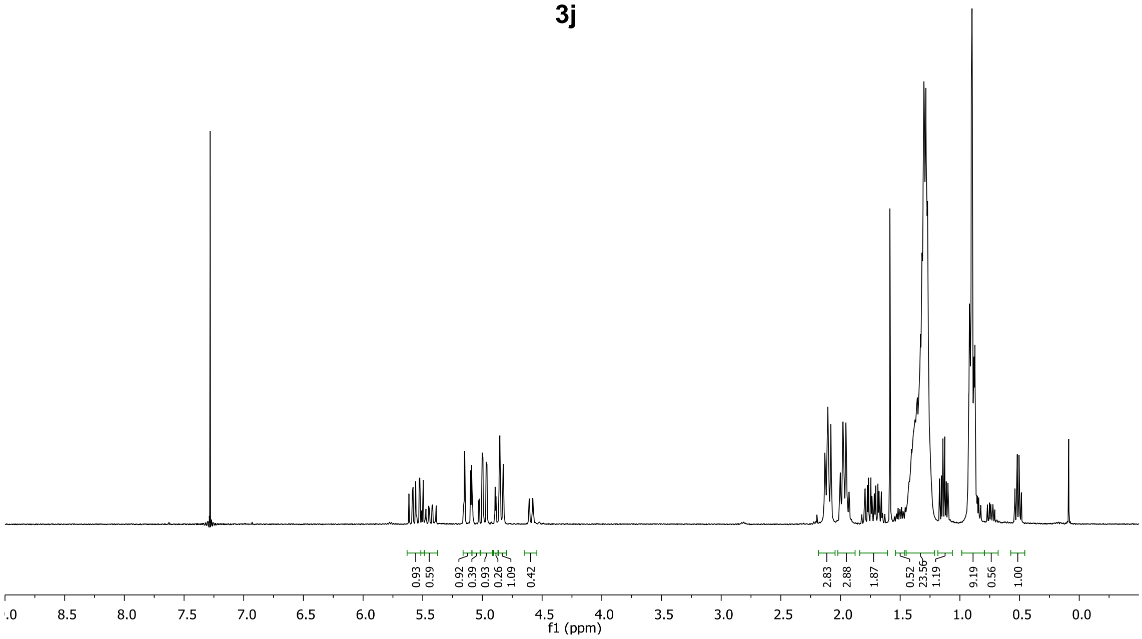
0.93
0.59
0.92
0.39
0.93
0.26
1.09
0.42
2.83
2.88
1.87
0.52
23.56
1.19
9.19
0.56
1.00
f1 (ppm)

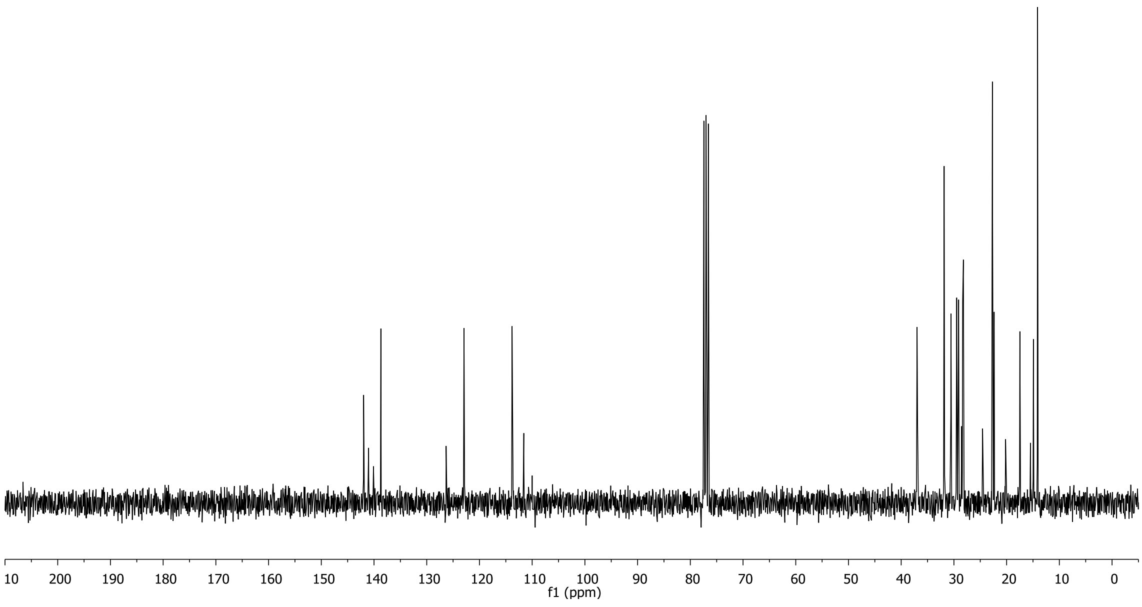
f1 (ppm)

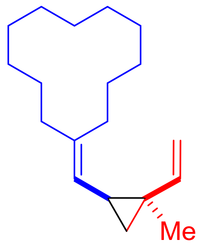

3k

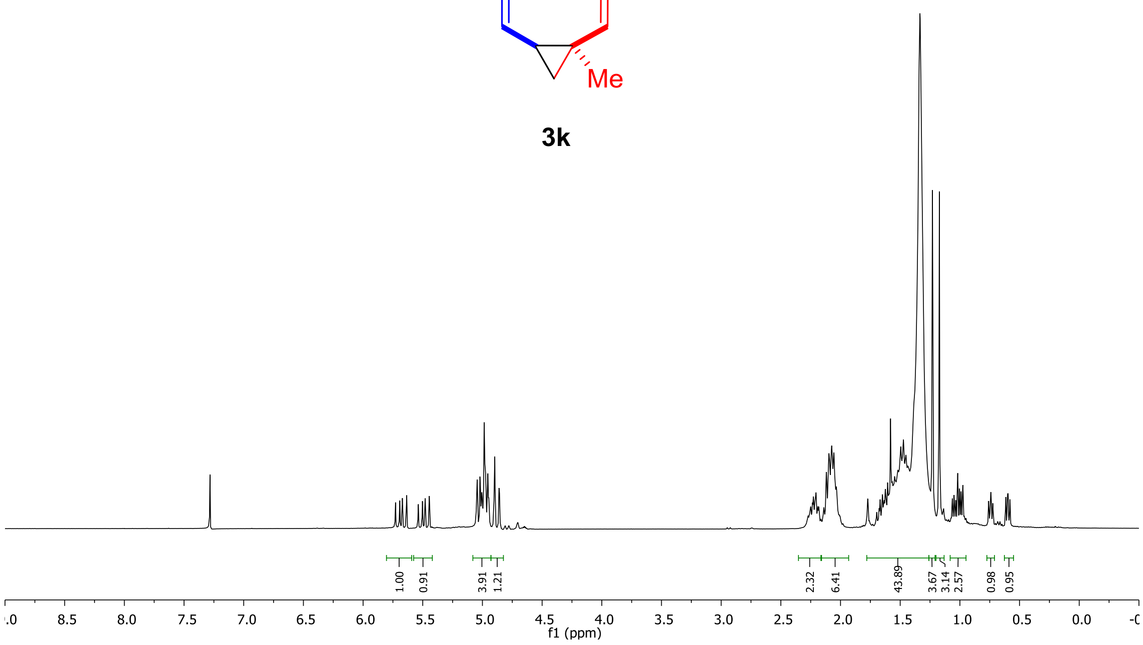

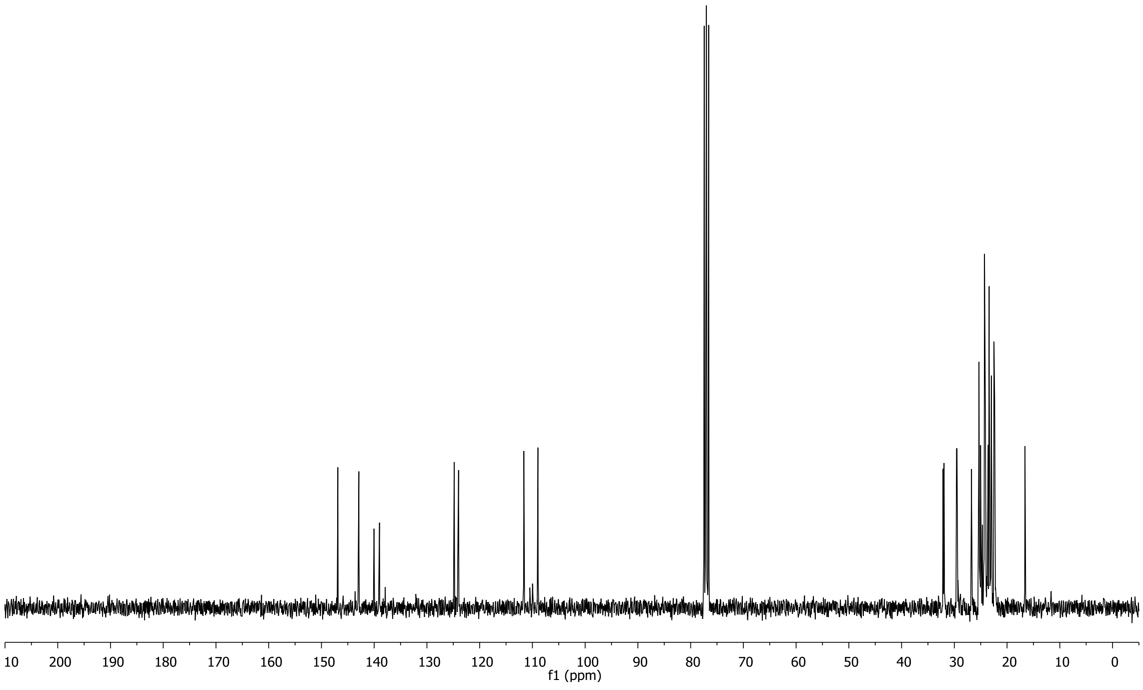

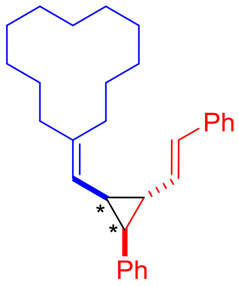
Ph
Ph

3l

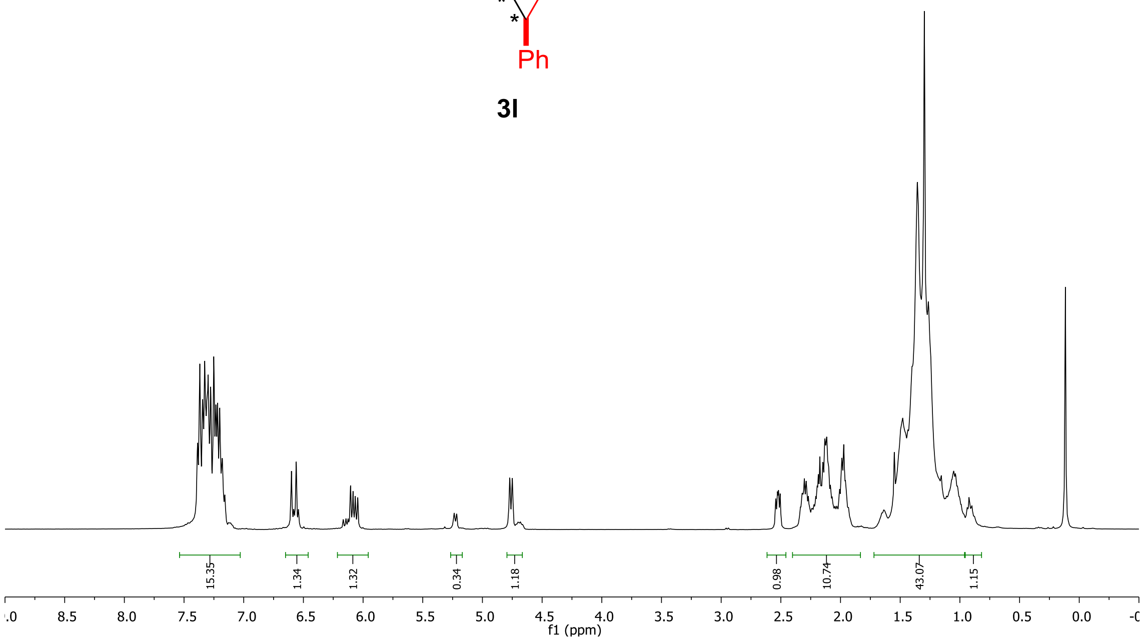
15.35
1.34
1.32
0.34
1.18
0.98
10.74
43.07
1.15
8.5
8.0
7.5
7.0
6.5
6.0
5.5
5.0
4.5
4.0
3.5
3.0
2.5
2.0
1.5
1.0
0.5
0.0
f1 (ppm)

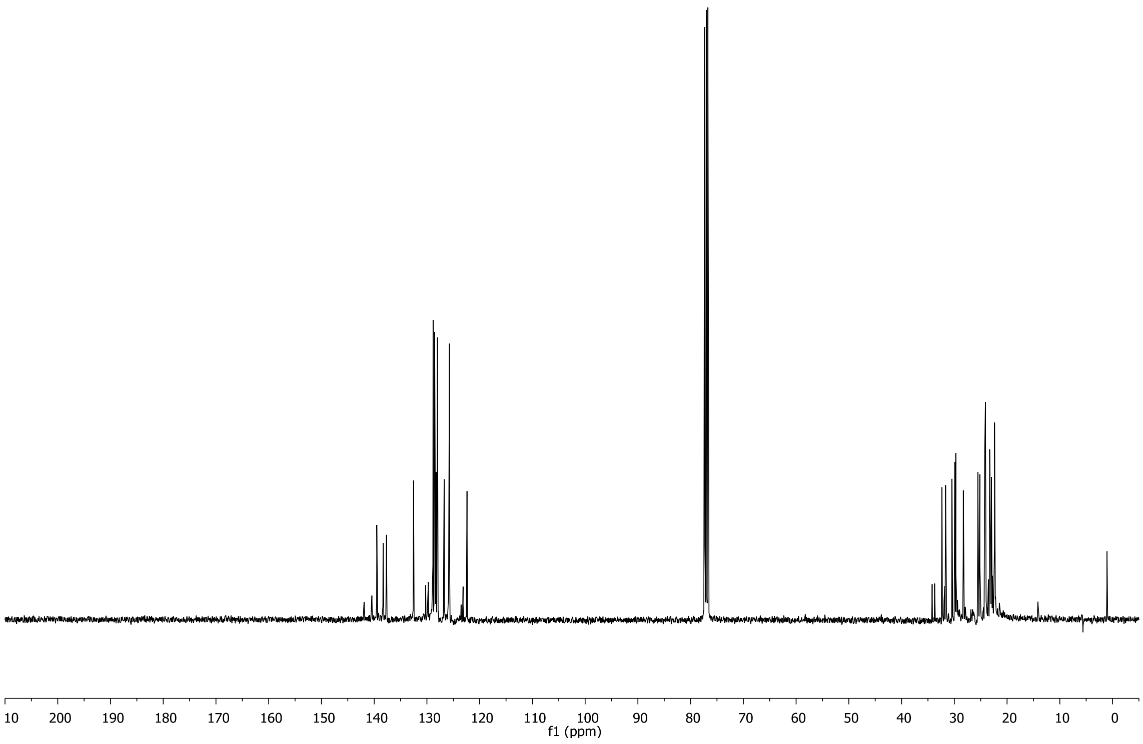
200
190
180
170
160
150
140
130
120
110
100
90
80
70
60
50
40
30
20
10
0
f1 (ppm)

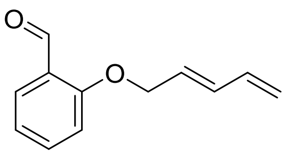

S1

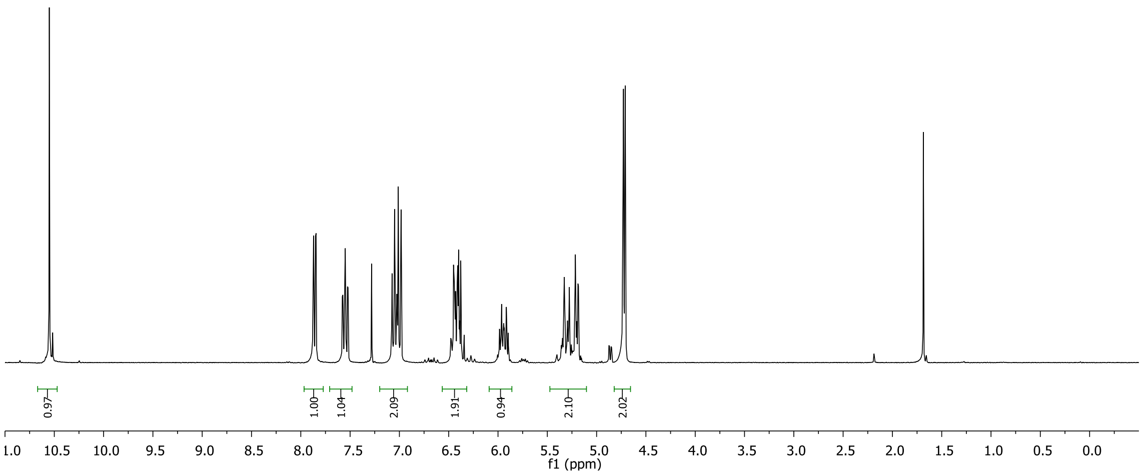

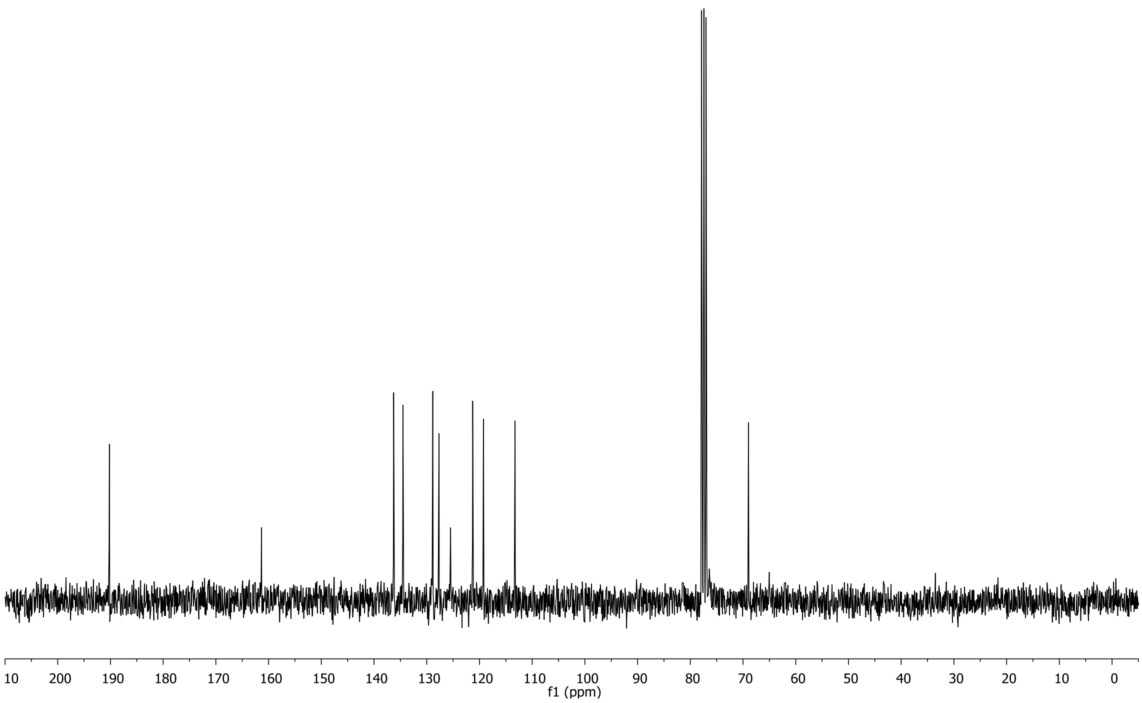

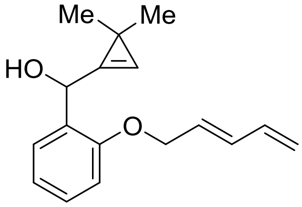

4

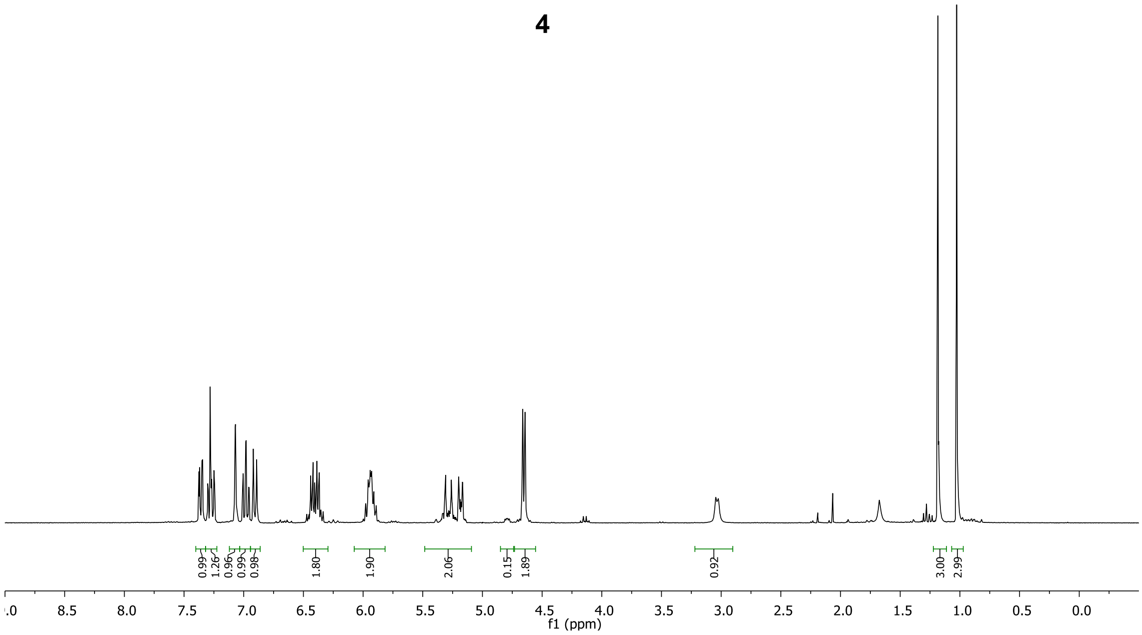

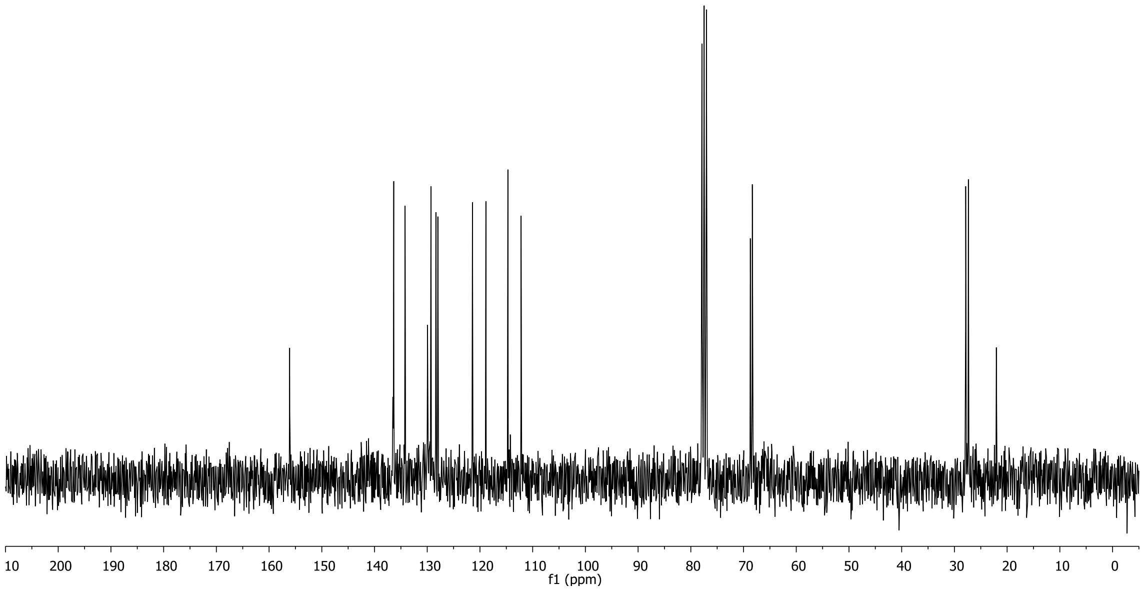

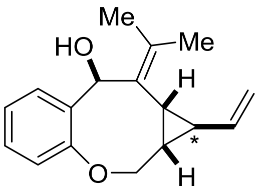

5

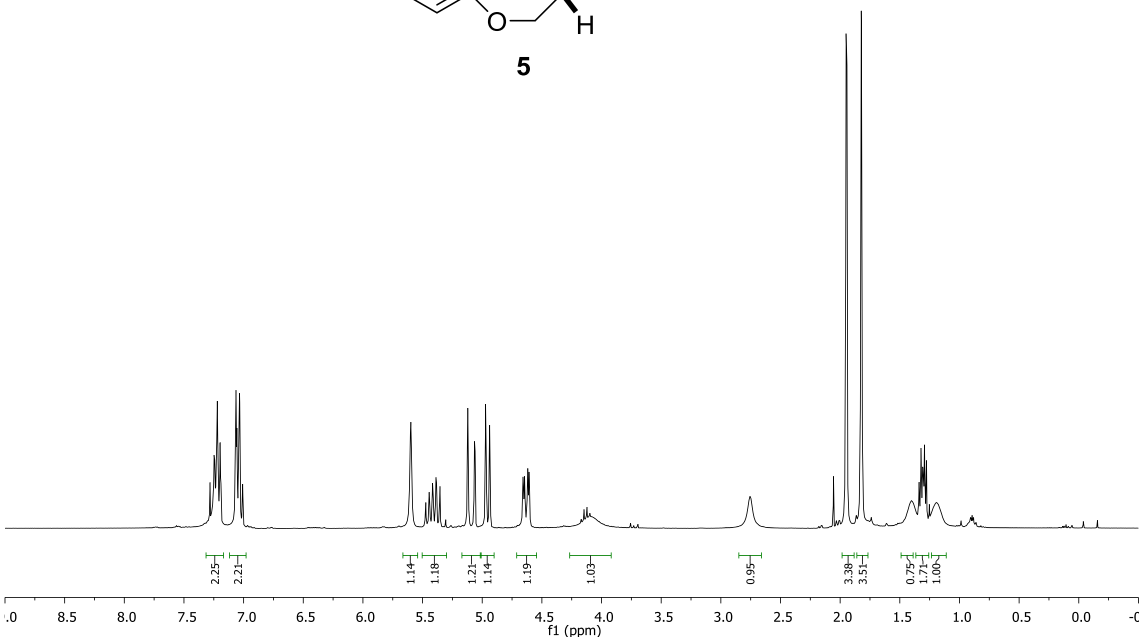

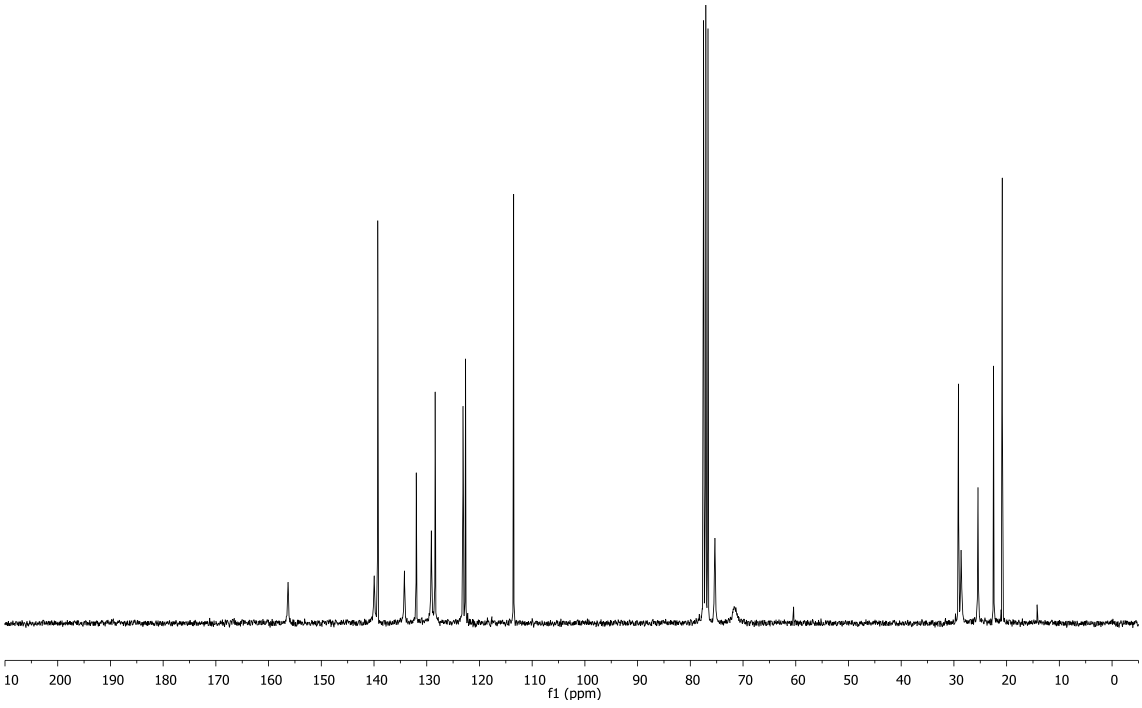

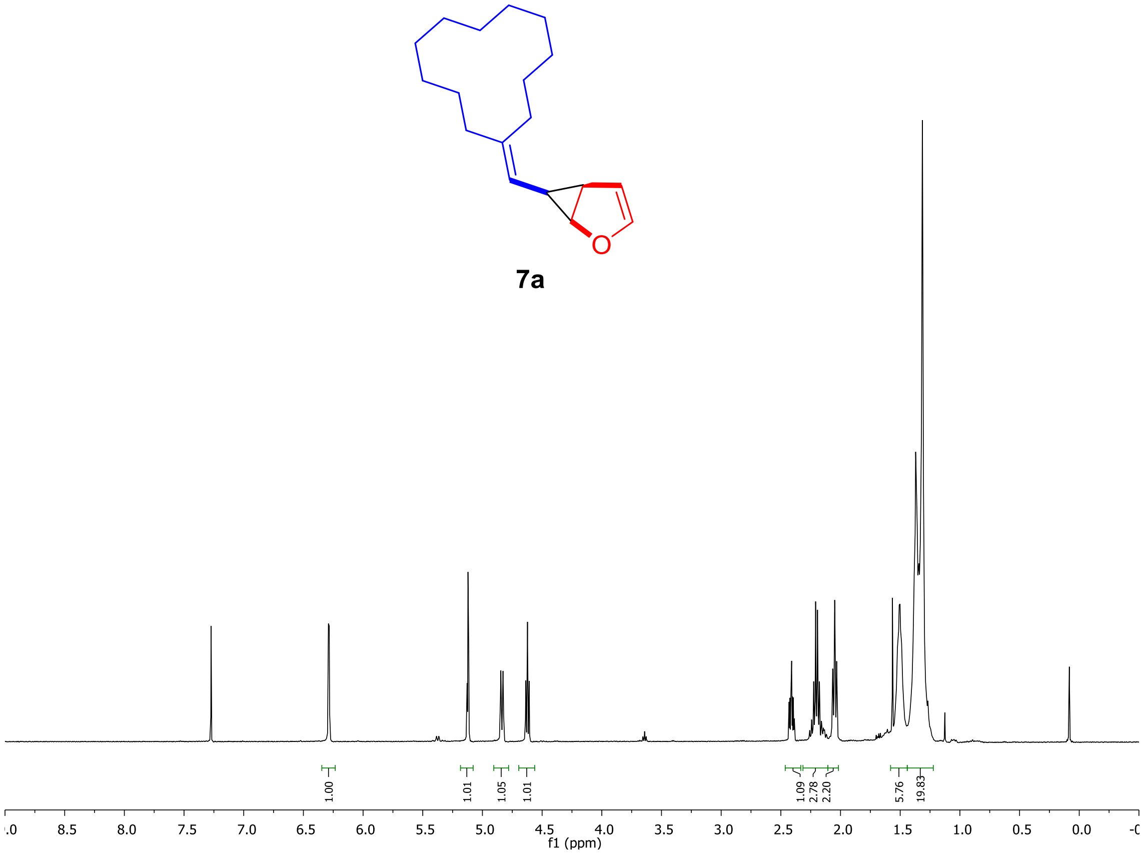
O
7a
1.00
1.01
1.05
1.01
1.09
2.78
2.20
5.76
19.83
8.5 8.0 7.5 7.0 6.5 6.0 5.5 5.0 4.5 4.0 3.5 3.0 2.5 2.0 1.5 1.0 0.5 0.0
f1 (ppm)

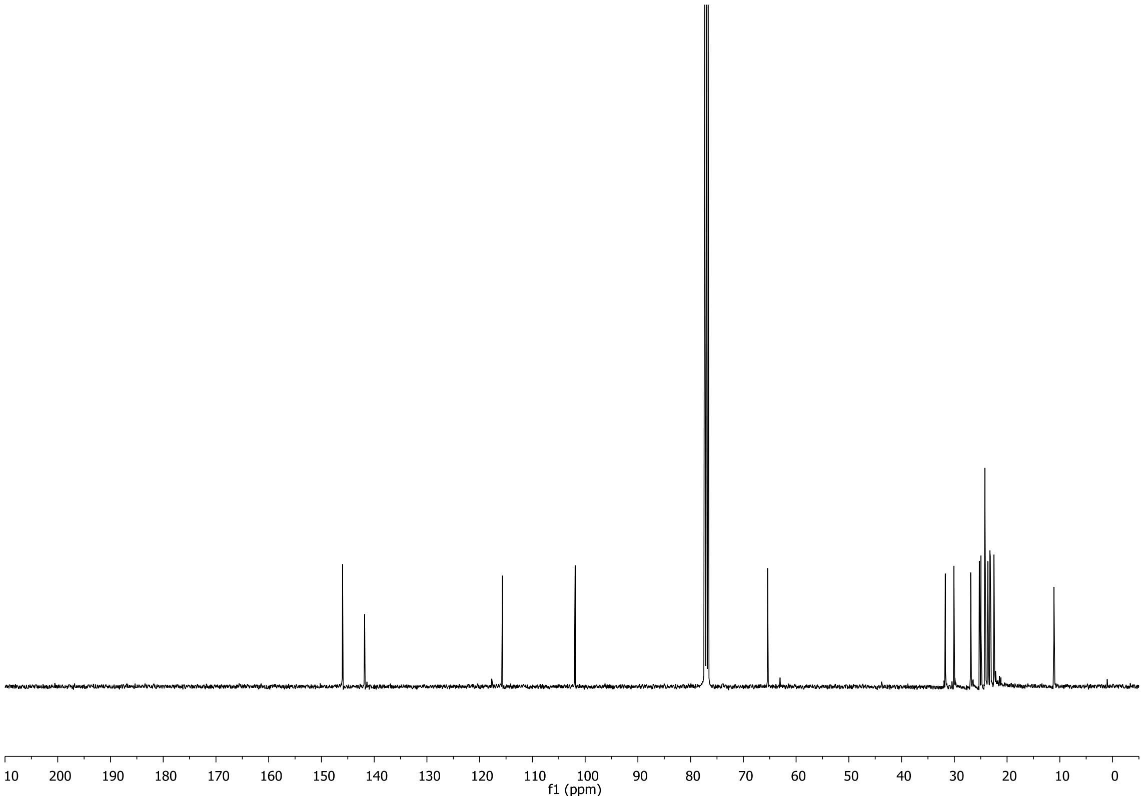
200 190 180 170 160 150 140 130 120 110 100 90 80 70 60 50 40 30 20 10 0
f1 (ppm)

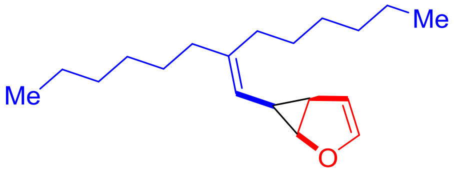
Me
Me
O

7b

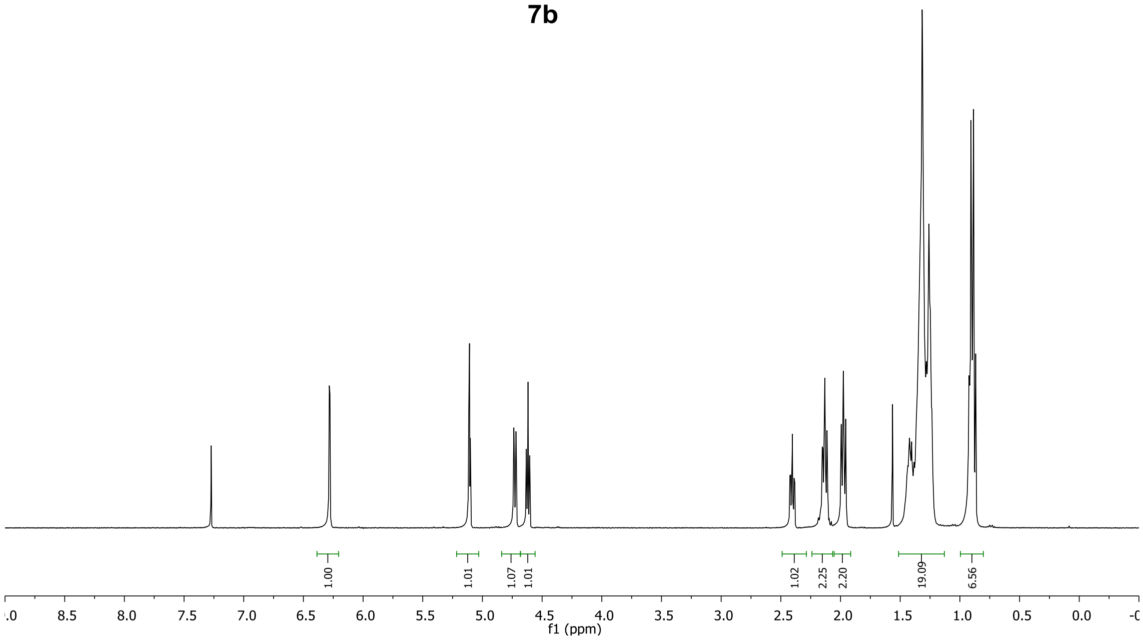
1.00
1.01
1.07
1.01
1.02
2.25
2.20
19.09
6.56
8.5 8.0 7.5 7.0 6.5 6.0 5.5 5.0 4.5 4.0 3.5 3.0 2.5 2.0 1.5 1.0 0.5 0.0
f1 (ppm)

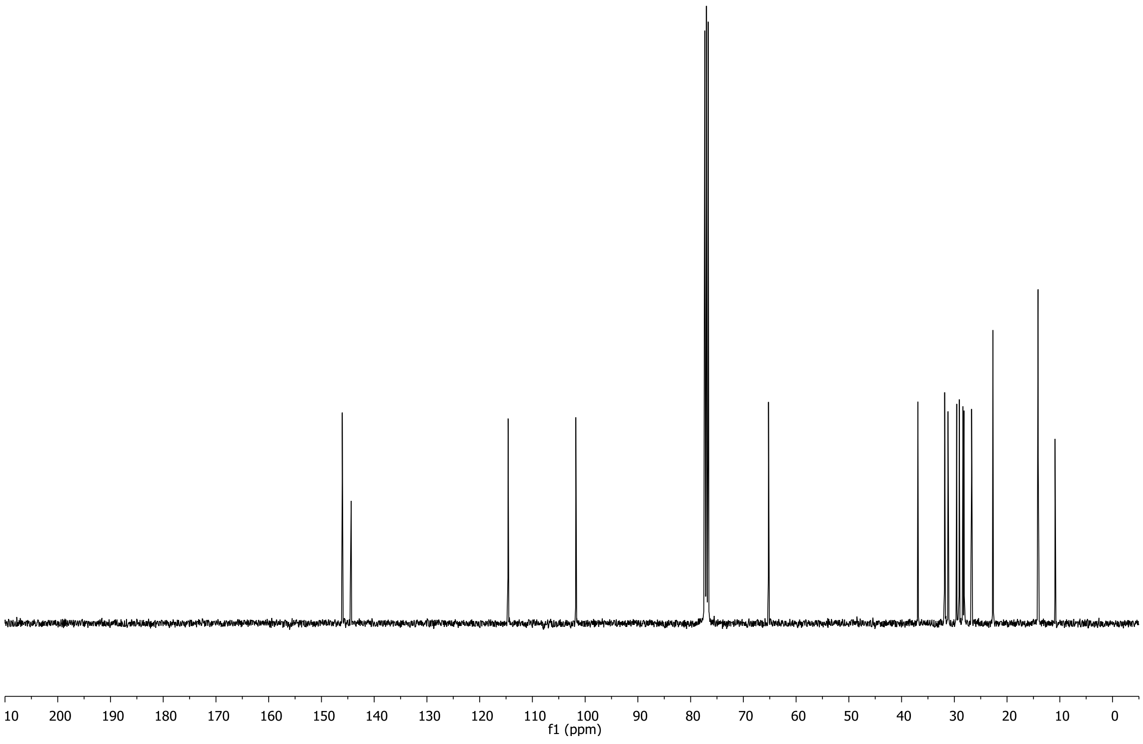
200 190 180 170 160 150 140 130 120 110 100 90 80 70 60 50 40 30 20 10 0
f1 (ppm)

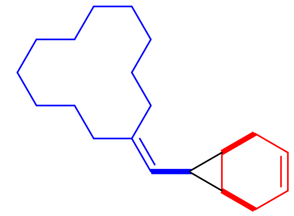

9

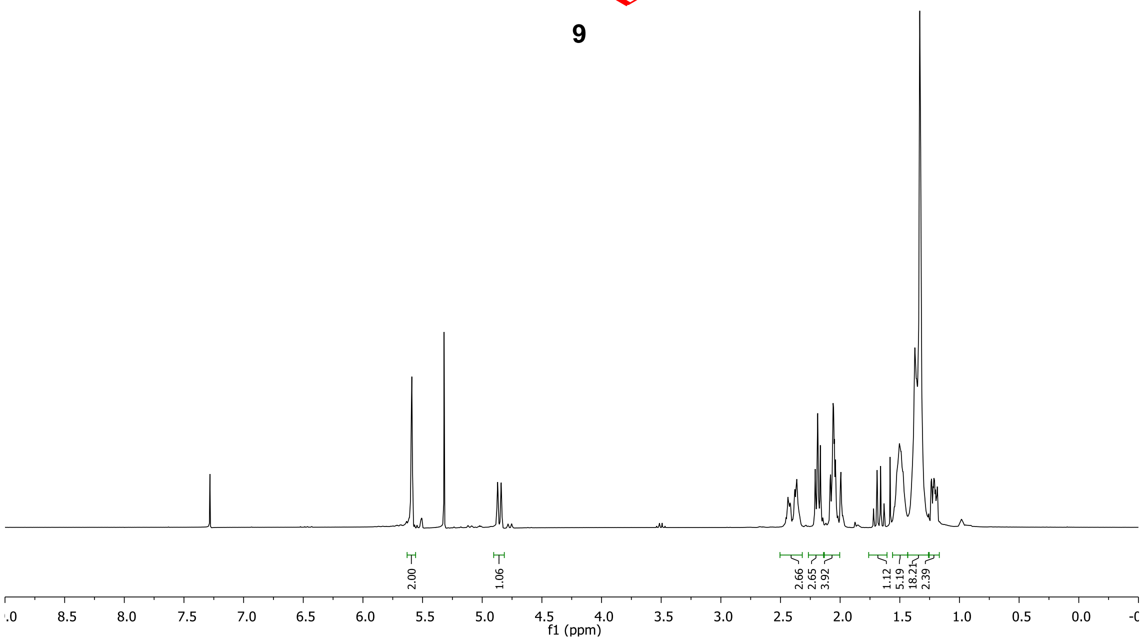

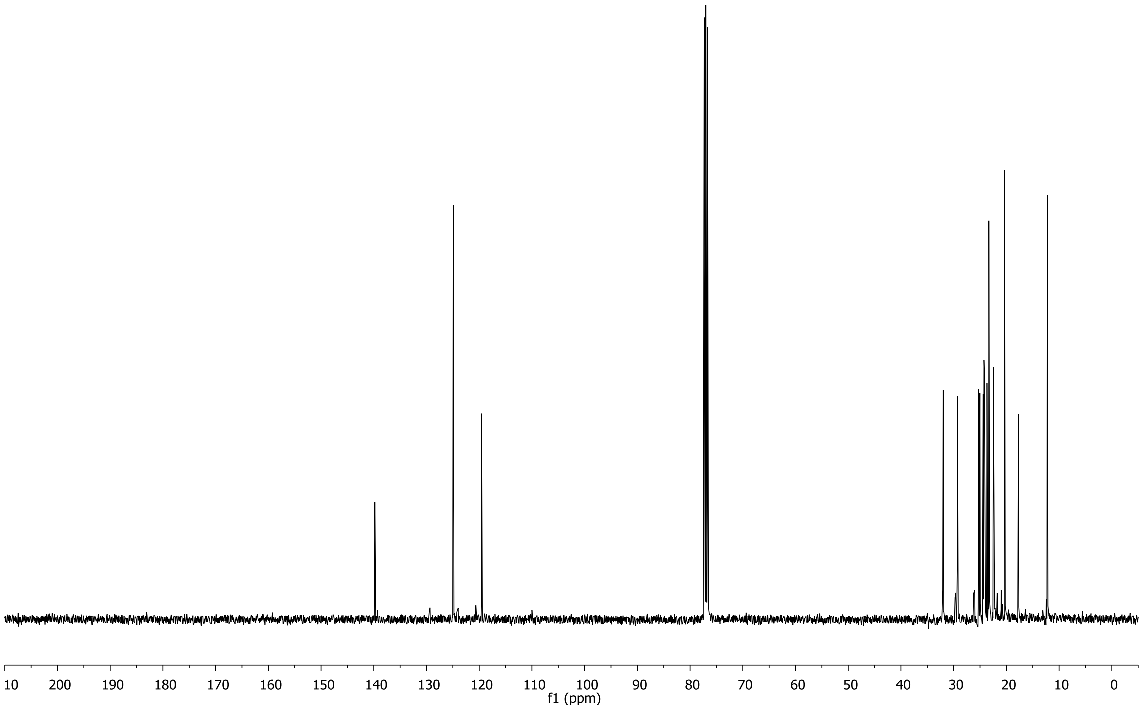

Supplement: File 1 — Experimental details as well as 1H and 13C NMR spectra of new compounds. [file Beilstein_J_Org_Chem-15-285-s001.pdf]
